# Profiles of Emotional Separation and Parental Trust from Adolescence to Emerging Adulthood: Age Differences and Associations with Identity and Life Satisfaction

**DOI:** 10.1007/s10964-022-01716-z

**Published:** 2022-12-16

**Authors:** Kazumi Sugimura, Shogo Hihara, Kai Hatano, Reiko Nakama, Satoko Saiga, Manabu Tsuzuki

**Affiliations:** 1 Graduate School of Humanities and Social Sciences, Hiroshima University, 1-1-1 Kagamiyama, Higashihiroshima, Hiroshima 739-8524 Japan; 2 Faculty of Business Administration, Matsuyama University, 4-2 Bunkyo-cho, Matsuyama, Ehime 790-8578 Japan; 3 Graduate School of Sustainable System Science, Osaka Metropolitan University, 1-1 Gakuen-cho, Naka-ku, Sakai, Osaka 599-8531 Japan; 4 Graduate School of Education, Hyogo University of Teacher Education, 942-1 Shimokume, Kato, Hyogo 673-1494 Japan; 5 Faculty of Letters, Chuo University, 742-1 Higashinakano, Hachioji, Tokyo, 192-0393 Japan

**Keywords:** Adolescence, Emerging adulthood, Parent–youth relationship, Identity, Life satisfaction, Japan

## Abstract

Youth become psychologically independent by emotionally separating from their parents and simultaneously developing a sense of trust in them. While these relational components have been addressed separately, studies focusing on the change in dynamics of these components are lacking. This study examined profiles of parent–youth relationship quality based on emotional separation and parental trust, age differences in the prevalence of these profiles, and age differences in the associations between the profiles, identity, and life satisfaction. Participants included 14,428 youth living in Japan from five age groups (44.8% girls/women; *M*_age_ = 20.6 years; range = 12–25 years). Six profiles were identified: *healthy–independent*, *unhealthy–independent*, *balanced*, *moderate/ambivalent*, *connected*, and *distant*. The *connected* profile was predominant among early adolescents, while the *healthy–independent* profile was predominant among late adolescents and early and middle emerging adults. Among all age groups, identity synthesis was the highest in the *healthy–independent* profile, and life satisfaction was the highest and identity confusion was the lowest in the *healthy–independent* and *connected* profiles. These findings indicate that young people navigate the process of becoming independent from their parents by balancing emotional separation and parental trust, and this balance relates to identity development and life satisfaction.

## Introduction

The quality of parent–youth relationship tends to become more emotionally separated, equal, and reciprocal during adolescence (for a review, see Branje, 2018); the process is characterized by the change in dynamics of various components of this relationship (for a review, see Koepke & Denissen, 2012). *Emotional separation* (Blos, 1967; separation–individuation theory) and *parental trust* (Bowlby, 1988; attachment theory) are key components of parent–youth relationship quality. Parental trust contributes positively to youth identity development and life satisfaction, whereas emotional separation is negatively associated with them among adolescents (Sugimura et al., 2018) and emerging adults (Žukauskienė et al., 2020) in both Europe and Japan. Despite these cumulative findings on the role of emotional separation and parental trust, prior studies only examined the separate roles of these relational components in youth psychosocial adjustment. They did not focus on the nuanced dynamics of emotional separation and parental trust represented by the profiles of these components. Therefore, the profiles that have been identified, the age differences that exist in the profiles, and how the profiles relate to psychosocial adjustment from adolescence to emerging adulthood are unclear. To address this research gap, this study aimed to identify profiles of emotional separation and parental trust and examine age differences in the profiles, as well as their associations with youth identity (i.e., synthesis and confusion) and life satisfaction from adolescence to emerging adulthood.

### Separation–Individuation and Attachment

Psychological independence from parents is an intricate process and has been explained in the separation–individuation theory (Blos, 1967) and attachment theory (Bowlby, 1988). Separation–individuation theory focuses on the process of emotional separation of the adolescent from parents. Emotional separation is defined as intrapsychic separation from parents, embracing two major subcomponents (Ferrer-Wreder & Kroger, 2020)—disengaging from the childhood representation of the parent (hereafter, *self–disengagement*) and recognizing the self and parents as separate individuals (hereafter, *self–other recognition*). However, independence from parents is not merely represented by emotional separation. Attachment theory emphasizes the importance of the emotional bond between parents and children, which contributes to the child’s adaptive independence from their parents—a process of separation accompanied by parental trust (for a review, see Allen & Tan, 2016). Parental trust refers to the feeling of security children derive from knowing that their parents understand and respect their needs and desires; this serves as a foundation on which children believe in their accessibility to parents in times of threat (Armsden & Greenberger, 1987).

The youth’s independence is a process of shifting the balance between emotional separation and parental trust. From the separation–individuation perspective, children enter the period of adolescence with low emotional separation (self–disengagement and self–other recognition); subsequently, they experience gradual disengagement from infantile parental representation, characterized as high self–disengagement. They use this self–disengagement as a necessary “stepping stone” (Beyers et al., 2003, p. 361) toward becoming fully independent from parents; they experience a more differentiated distinction between themselves and parents in emerging adulthood (Koepke & Denissen, 2012), which may be represented by self–other recognition. From the attachment perspective, these changes can be explained by the decreasing parental trust and the increasing importance of peers and romantic partners as sources of support (Oudekerk et al., 2015). Although parental trust is restrained as youth get older (Branje, 2018), it continues serving as a base on which they can explore the broader world outside the family, safely separate from their parents (Allen & Tan, 2016). This suggests the importance of capturing the youth’s independence from the perspectives of multiple relational components (profiles). However, prior research has addressed emotional separation and parental trust separately (e.g., Sugimura et al., 2018), and no studies have examined changes in the balance between emotional separation and parental trust using profiles.

### Profiles of Emotional Separation and Parental Trust, and the Age Differences

Not all children, at all times, experience a *single* type of balance of these relational components. It is logical to assume that there are *diverse* profiles of self*–*disengagement, self–other recognition, and parental trust. Identifying these profiles is important to gain insight into practical implications regarding the types of youth who are at risk and need to be supported to attain healthy independence from their parents.

To understand such diverse relationship qualities, this study adopted a person-centered approach (von Eye & Bogad, 2006), which allowed us to examine relationship quality from the perspective that there are different populations regarding a balance between self–disengagement, self–other recognition, and parental trust. Thus, this approach is particularly suitable to clarify heterogeneity in the dynamics of key relational components in different profiles by combining these components.

Based on the separation–individuation and attachment theories, there are logically assumed diverse profiles that create a unique quality of independence. High parental trust with low self–disengagement and low self–other recognition represents a strong emotional connection between parents and youth, in which youth experience *less independence*. Contrastingly, high parental trust with high self–disengagement and/or high self–other recognition illustrates *healthy independence*, in which youth exert their own decisions and intentions while maintaining a connection with parents. Several interim profiles may lie between these two profiles. For instance, low parental trust with high self–disengagement and high self–other recognition implies a distant type of independence, whereas low parental trust with high self–disengagement and low self–other recognition indicates a radical type of independence and may produce a conflictual, unhealthy quality of independence.

Moreover, as reviewed in the previous section, separation–individuation and attachment theories suggest age differences in profiles; the less independent (or connected) profile would be prevalent in younger age groups, whereas the healthy–independent profile would be common in older age groups. However, there is a lack of empirical research on age differences in profiles of emotional separation and parental trust from adolescence to emerging adulthood. Thus far, only one study has examined age trends in profiles combining relational components, albeit investigating different components from those used in the current study (support, negative interaction, and power; Hadiwijaya et al., 2017). Their findings implied that younger adolescents (aged 12–16 years) moved away from parental authority and experienced increased conflict, whereas older adolescents (aged 16–20 years) improved their relationships with parents. However, no studies have examined independence in adolescence and emerging adulthood from a combination of emotional separation and parental trust perspectives. The current study sought to reveal the process of becoming independent from parents—via emotional separation and parental trust—from adolescence to emerging adulthood.

### Relationships between Profiles and Psychosocial Adjustment

Secure parent–adolescent relationships can foster healthy autonomy and in turn, positive psychosocial adjustment (Allen & Tan, 2016). Accordingly, this study focused on a sense of identity (synthesis and confusion)—a developmental facet (Sugimura et al., 2018)—and life satisfaction—a well-being facet (Žukauskienė et al., 2020)—as crucial indicators of youth psychosocial adjustment. Identity synthesis refers to the extent that various aspects of one’s identity fit together, representing a sense of recognition and feeling that one knows where one is headed. In contrast, identity confusion represents feeling confused as to what one is doing in life, along with being unable to implement and maintain lasting commitments to important life choices (Erikson, 1968). Life satisfaction represents the cognitive dimension of subjective well-being and refers to the global assessment of a person’s quality of life according to their own chosen criteria (Diener et al., 1985).

Studies have demonstrated the separate roles of emotional separation and parental trust in identity and life satisfaction. Emotional separation (focusing on self–disengagement indicating a conflictual relationship with parents) hindered identity synthesis, while parental trust contributed to it in adolescents in both Europe (Italy and Lithuania) and Japan (Sugimura et al., 2018). Moreover, parental trust benefited life satisfaction in emerging adulthood in Europe (Lithuania) and Japan (Žukauskienė et al., 2020), in line with the theoretical notion that it is a secure base for managing external world challenges (Allen & Tan, 2016). Thus, although no study has addressed the profiles of emotional separation and parental trust, it is possible that the connected and healthy–independent profiles represented by high parental trust would show high levels of identity synthesis and life satisfaction and low levels of identity confusion.

Nevertheless, as youth face different expectations from society (e.g., age-based standards) as they age, it is possible that profiles associated with greater psychosocial adjustment are different across age groups. For instance, a study revealed that disengaging from parents too early, around the time of puberty, resulted in serious consequences for adolescent maladjustment (Dishion et al., 2004). As adolescents in this age group have not yet fully developed self-regulation (Branje, 2018), they still need to be guided by their parents. This implies that the connected profile represented by high parental trust may be preferable for psychosocial adjustment among younger age groups. In middle and late adolescence, being aware of the distinctiveness between one’s own and one’s parents’ perspectives plays a critical role in psychosocial adjustment (Grotevant & Cooper, 1985). Furthermore, in emerging adulthood, youth establish more mature connections with their parents (Koepke & Dennissen, 2012). Therefore, the healthy–independent profile, represented by both high parental trust and high emotional separation, would be associated with greater psychosocial adjustment in older age groups.

### The Japanese Context

Contemporary Japanese youth live in a unique setting that encompasses both individualism and collectivism. Traditionally, parent–child relationships in Japan have been characterized by their strong emotional ties from infancy through adulthood (Rothbaum et al., 2000). This emotional closeness between parents and children is usually maintained in adolescence when young people explore the world beyond family relationships. For instance, in Japan, exploration opportunities owing to separating from parents are not as valued as in Western cultures (Umemura et al., 2018). However, while some of these values of closeness are maintained in Japanese society and among older generations, younger generations are increasingly valuing autonomy and agency (Sugimura, 2020). Therefore, it can be assumed that Japanese youth become independent from parents as suggested by the separation–individuation and attachment theories—models developed in the Western contexts. Thus, Japanese youth is an appropriate example of how young people become independent from parents while maintaining strong emotional bonds with them, and in turn, how this relates to healthy identity and life satisfaction. The findings may provide insights into the process of becoming independent from parents in collectivistic Asian cultures aside from Japan.

## Current Study

Although emotional separation and parental trust are not independent concepts, and the balance of each leads to healthy independence for youth, previous studies have examined them separately (e.g., Sugimura et al., 2018). Moreover, their relationship with youth’s psychosocial adjustment is unclear. This study aimed to fill these gaps by identifying diverse profiles of parent–youth relationship quality and investigating differences in the prevalence of these profiles and their associations with identity and life satisfaction across age groups. The current study adopted a person-centered approach, targeting Japanese youth from five age groups: early adolescence (12–13 years), middle adolescence (15–16 years), late adolescence (18–19 years), early emerging adulthood (21–22 years), and middle emerging adulthood (24–25 years). Three research objectives were defined.

First, this study explored whether various profiles of parent–youth relationship quality exist, combining three components: self–disengagement, self–other recognition, and parental trust (Objective 1). Based on the theories on the optimal balance between emotional separation and parental trust (Allen & Tan, 2016), some youth would belong to the profile characterizing less independence (Hypothesis 1a) and some to that characterizing healthy independence (Hypothesis 1b). Between these two profiles, there would also be some interim profiles (Hypothesis 1c); however, specific predictions were not made owing to a lack of evidence.

Second, this study examined differences in the prevalence of the identified profiles across five age groups (Objective 2). Based on the theoretically suggested trends of change in the balance between emotional separation and parental trust (Koepke & Dennissen, 2012), the percentage of individuals in the profiles represented by high parental trust would be high in the early and middle adolescence groups (Hypothesis 2a), whereas that in the profiles represented by high self–disengagement and/or self–other recognition with high parental trust would be high in the late adolescence and early and middle emerging adulthood groups (Hypothesis 2b).

Third, this study investigated age differences in the associations between relationship profiles and identity synthesis, identity confusion, and life satisfaction (Objective 3). Based on a literature review (e.g., Dishion et al., 2004), in younger age groups (early and middle adolescence), the profiles characterized by high parental trust would yield high scores on identity synthesis and life satisfaction, and low scores on identity confusion (Hypothesis 3a); whereas, in older age groups (late adolescence and early and middle emerging adulthood), profiles characterized by high self–other disengagement and/or self–other recognition with high parental trust would score high on identity synthesis and life satisfaction, and low on identity confusion (Hypothesis 3b).

## Method

### Participants

Participants were 14,428 adolescents and youth living in Japan, including 1739 early adolescents (41.6% girls), 1803 middle adolescents (32.4% girls), 1609 late adolescents (60.6% girls), 4641 early emerging adults (61.1% women), and 4636 middle emerging adults (61.1% women). The present sample was taken from the International Adolescent Psychology Project in Japan (http://web.hyogo-u.ac.jp/nakama/iappj/src/index.html; Hatano et al., 2022).

The early adolescent participants were junior high school students from middle- to high-academic level schools (72.6% from public schools and 27.4% from private schools). They resided in relatively urban areas in Japan (Kansai and Kanto districts). The average age was 12.7 (*SD* = 0.5) years.

The middle adolescent participants were high school students from middle- to high-academic level schools (45.9% from public schools; 54.1% from private schools). They resided in relatively urban areas in Japan (Kansai and Kanto districts). The mean age was 15.7 (*SD* = 0.5) years.

The late adolescent sample comprised 85.3% students (67.9% university students, 5.0% junior college students, 10.9% technical students, and 1.5% others), 8.8% workers, 4.0% unemployed people, and 1.9% others. Among them, university students were from middle- to high-academic level universities. Regarding the annual household incomes of late adolescent participants who were not university students, 2.9, 5.2, and 1.0% had low (<2 million yen), middle (2–8 million yen), and high (>8 million yen) income levels, respectively; income levels were unknown for 90.9% of the sample. Regarding geographical region, 91.4% lived in relatively urban areas (Kansai, Kanto, and Chubu districts), 8.4% lived in relatively rural areas (Hokkaido, Tohoku, Chugoku, Shikoku, and Kyushu districts), and 0.2% did not provide relevant information. The mean age was 18.8 (*SD* = 0.4) years.

The early emerging adult sample consisted of 60.1% students (58.1% university students, 0.2% junior college students, 1.6% technical students, and 0.2% others), 26.1% workers, 10.6% unemployed people, and 3.1% others. Regarding household income per year, 19.7, 28.1, and 8.0% had low (<2 million yen), middle (2–8 million yen), and high (>8 million yen) income, respectively; income levels were unknown for 44.2% of the sample. Regarding residential areas, 70.2% lived in relatively urban areas (Kansai, Kanto, and Chubu districts) and 29.8% lived in relatively rural areas (Hokkaido, Tohoku, Chugoku, Shikoku, and Kyushu districts). The mean age was 21.8 (*SD* = 0.4) years.

The middle emerging adult sample included 8.3% students (3.3% university students, 0.04% junior college students, 0.9% technical students, and 4.1% others), 69.8% workers, 17.1% unemployed people, and 4.8% others. Regarding household income per year, 11.0, 48.9, and 9.3% had low (<2 million yen), middle (2–8 million yen), and high (>8 million yen) income, respectively; income levels were unknown for 30.9% of the sample. Regarding residential areas, 69.7% lived in relatively urban areas (Kansai, Kanto, and Chubu districts), and 30.3% lived in relatively rural areas (Hokkaido, Tohoku, Chugoku, Shikoku, and Kyushu districts). The mean age was 24.8 (*SD* = 0.4) years.

Overall, the study sample was characterized by middle socioeconomic status in terms of both academic level and household income. Moreover, most participants resided in the Kanto, Chubu, and Kansai districts, which are large urban centers in Eastern, Central, and Western parts of Japan, respectively. These areas are more populated and have greater income inequality, more educational institutions (schools and universities), and higher tertiary education enrollment rate than other areas (Hatano et al., 2022).

To examine the distributions of gender and residential areas among age groups, chi-square tests were conducted. Gender distributions differed significantly by age groups, χ^2^ (4, *N* = 14,418) = 655.58, *p* < 0.001, Cramer’s *V* = 0.21, *p* < 0.001. Girls/women were underrepresented in the early and middle adolescent groups and overrepresented in the late adolescent and early and middle emerging adult groups. Furthermore, the distributions of residential areas differed significantly by age groups, χ^2^ (4, *N* = 14,425) = 1585.59, *p* < 0.001, Cramer’s *V* = 0.33, *p* < 0.001. Those living in relatively urban areas were overrepresented in the early and middle adolescent groups and underrepresented in the late adolescent and early and middle emerging adult groups. Thus, since influences of gender and residential areas may exist, the results of the analyses are discussed considering these influences (see more details of the sensitivity analyses in the Results section).

### Procedure

For early and middle adolescents, data were collected at 10 schools. The authors requested and received permission from the school authorities to conduct the survey. Early and middle adolescents responded to paper-and-pencil questionnaires during class time. For late adolescents, the data were collected in two ways. For university students, flyers containing a hyperlink to the web-based survey hosted on Creative Survey (https://jp.creativesurvey.com/) were distributed in classrooms at 60 universities. The remaining participants (e.g., technical school students and workers) were recruited from a participant pool of an online survey company, Kanden CS Forum (https://www.kcsf.co.jp/?p=home). Early and middle emerging adults were sampled from a participant pool of the same online research company. People of various age groups, professions, and regions around Japan are registered with this company. Registrants of the research company who passed the company’s quality control (e.g., periodically checking for the honesty of participants’ answers, irregularities in response styles, and updates on demographic information) received an email detailing the study purpose. Those who were willing to participate received a hyperlink to the online questionnaires. For participation, university students in late adolescence received reward points equivalent to 500 JPY (approximately 5 USD), and other late adolescents and early and middle emerging adults received reward points equivalent to 40 JPY (approximately 0.4 USD).

### Measures

#### Parent–youth relationship

Assessment of the parent–youth relationship included self–disengagement, self–other recognition, and parental trust.

##### Self–disengagement

This was measured using the Emotional Separation Scale (Beyers et al., 2005; see Sugimura et al., 2018, for the Japanese version). This scale comprises 12 items (sample item: “There are some things about me that my parents don’t know”) rated on a four-point scale, ranging from *strongly disagree* (1) to *strongly agree* (4). Cronbach’s alphas for the scale were 0.82, 0.82, 0.84, 0.83, and 0.83 for early adolescents, middle adolescents, late adolescents, early emerging adults, and middle emerging adults, respectively.

##### Self–other recognition

This was assessed using the Psychological Independence Scale (Mizumoto & Yamane, 2011), which was originally developed and validated for use with Japanese-speaking samples based on Blos’ (1967) conceptions, including items assessing recognition of the self and parents as separate individuals. The scale consists of five items (sample item: “My lifestyle differs from that of my parent;” see the Appendix for all five items) rated on a five-point scale, ranging from *strongly disagree* (1) to *strongly agree* (5). Cronbach’s alphas were 0.68, 0.68, 0.69, 0.73, and 0.74 for early adolescents, middle adolescents, late adolescents, early emerging adults, and middle emerging adults, respectively.

##### Parental trust

This was assessed using the Parental Trust subscale in the short version of the Inventory of Parent and Peer Attachment (Nada-Raja et al., 1992; see Sugimura et al., 2018, for the Japanese version). This subscale includes four items (sample item: “My parents respect my feelings”) rated on a four-point scale, ranging from *strongly disagree* (1) to *strongly agree* (4). Cronbach’s alphas were 0.82, 0.82, 0.82, 0.82, and 0.83 for early adolescents, middle adolescents, late adolescents, early emerging adults, and middle emerging adults, respectively.

#### Identity synthesis and confusion

Identity was assessed using the Identity subscale of Erikson’s Psychosocial Stage Inventory (Rosenthal et al., 1981; see Sugimura et al., 2016, for the Japanese version), which includes 12 items: six for identity synthesis (sample item: “I have got a clear idea of what I want to be”) and six for identity confusion (sample item: “I change my opinion of myself a lot”). One of the synthesis items, “I have a strong sense of what it means to be female/male,” was removed since it was regarded as undesirable to measure gender. Consequently, participants responded to five synthesis items and six confusion items using a five-point scale, ranging from *strongly disagree* (1) to *strongly agree* (5). Cronbach’s alphas were 0.68, 0.69, 0.73, 0.76, and 0.77 for synthesis, and 0.67, 0.64, 0.74, 0.75, and 0.75 for confusion, in samples of early adolescents, middle adolescents, late adolescents, early emerging adults, and middle emerging adults, respectively.

#### Life satisfaction

The Satisfaction with Life Scale (Diener et al., 1985) was used to measure life satisfaction. This comprises five items (sample item: “In most ways, my life is close to my ideal”) reported on a seven-point scale, ranging from *completely untrue* (1) to *completely true* (7). Cronbach’s alphas were 0.85, 0.84, 0.87, 0.87, and 0.74 for early adolescents, middle adolescents, late adolescents, early emerging adults, and middle emerging adults, respectively.

Among these measures, the alpha coefficients of the scales for self–other recognition and identity were below 0.70 in younger groups. Regarding self–other recognition, the coefficients are consistent with the value in a study on Japanese emerging adults (0.65; Mizumoto & Yamane, 2011). Regarding identity (both synthesis and confusion), the coefficients were consistent with those in previous studies on Japanese, Belgian, and American adolescents (0.62–0.87; Bogaerts et al., 2021; Meca et al., 2017; Sugimura et al., 2018). The results of this study are discussed considering the relatively low alpha coefficients.

### Missing Data

The completion rate of the survey was high (5.38% missing data). The Little’s (1988) Missing Completely at Random (MCAR) test indicated a significant result (χ^2^ (56,115) = 60,141.74, *p* < 0.001), with a normed χ^2^ (χ^2^/*df*) of 1.07. According to Bollen’s (1989) guidelines, this suggests that the data were likely missing at random. To address missing values, multiple imputation was performed using the SPSS 25.0 (Arbuckle, 2016). This replaced missing data with a 25-iteration setting and produced 20 multiple imputed datasets. Statistical analyses were performed on each imputed dataset and then combined to yield a single set of pooled results. The R 4.2.1 was also used to calculate pooled statistics for several analyses that cannot be reported by the SPSS (e.g., χ^2^ and *F* values). For statistics that could not be combined into a single pooled result by the SPSS or the R (e.g., adjusted standardized residuals), results obtained from one of the twenty imputed datasets were reported; these results are consistent with those from the remaining 19 datasets (see Online Resource 3).

### Analytic Plan

As a preliminary step, measurement invariance among the study variables was tested across age groups by a series of multigroup confirmatory factor analyses. Means, standard deviations, and correlations of the study variables were calculated.

To identify the profiles of parent–youth relationship quality (Hypothesis 1), Gore’s (2000) two-step cluster analysis^1^ was performed. In the first step, a series of hierarchical cluster analyses were conducted, employing Ward’s method on the basis of squared Euclidean distance. Two- to seven-cluster solutions were compared to select the number of clusters, using the following criteria: (1) theoretical meaningfulness of clusters, (2) parsimony, and (3) explanatory power (the cluster solution must explain more than 50% of the variance in the three types of parent–youth relationship). In the second step, iterative k-means cluster analysis was conducted with the selected number of clusters.

Age differences in the distributions of relationship profiles (Hypothesis 2) were tested through chi-square tests. Furthermore, to examine age differences in the association of relationship profiles with psychosocial adjustment (Hypothesis 3), a series of analyses of variance (ANOVAs) was performed, including the relationship profile, age, and relationship profile × age interaction as the independent variables and identity synthesis, identity confusion, and life satisfaction as the dependent variables.

Additional sensitivity analyses were conducted to examine whether gender and residential area moderate the results of the main analyses (age differences in the distributions of relationship profiles and associations between the relationship profiles and psychosocial adjustment). Dummy-coded gender (0 = boys/men, 1 = girls/women) and residential area (0 = relatively rural areas, 1 = relatively urban areas) were included as moderators.

## Results

### Preliminary Analyses

Table S1 presents the results of the measurement invariance tests. Regarding the parent–youth relationship, partial scalar invariance across age groups was established for self–disengagement, self–other recognition, and parental trust. Furthermore, partial scalar invariance across age groups was found for identity synthesis and identity confusion, as well as for life satisfaction (see more details in Online Resource 1).

Table 1 displays the means and standard deviations of the study variables for the five age groups. Regarding self–disengagement, middle adolescents and early and middle emerging adults scored the highest, followed by late and early adolescents. Regarding self–other recognition, early and middle emerging adults scored the highest, followed by late, middle, and early adolescents. Regarding parental trust, early, middle, and late adolescents scored higher than early and middle emerging adults.

**Table 1:** Descriptive statistics and age group differences

Variables		Total	Early adolescents	Middle adolescents	Late adolescents	Early emerging adults	Middle emerging adults	*F*-values	η^2^
Self–disengagement	*M* (*SD*)	2.79 (0.48)	2.61 (0.49)^a^	2.81 (0.42)^b^	2.76 (0.47)^c^	2.84 (0.48)^b^	2.83 (0.48)^b^	*F* (4, 21,654.77) = 84.55***	0.024
	α	0.83	0.82	0.82	0.84	0.83	0.83		
Self–other recognition	*M* (*SD*)	3.73 (0.69)	3.27 (0.74)^a^	3.72 (0.64)^b^	3.76 (0.65)^b,c^	3.81 (0.65)^c,d^	3.83 (0.66)^d^	*F* (4, 10,220.75) = 241.08***	0.065
	α	0.72	0.68	0.68	0.69	0.73	0.74		
Parental trust	*M* (*SD*)	2.96 (0.68)	3.01 (0.66)^a^	3.03 (0.63)^a^	3.02 (0.67)^a^	2.93 (0.68)^b^	2.93 (0.69)^b^	*F* (4, 22,676.92) = 13.05***	0.004
	α	0.82	0.82	0.82	0.82	0.82	0.83		
Identity synthesis	*M* (*SD*)	3.06 (0.78)	3.12 (0.77)^a^	3.13 (0.73)^a^	3.10 (0.78)^a^	3.02 (0.80)^b^	3.02 (0.78)^b^	*F* (4, 20,486.59) = 12.03***	0.003
	α	0.74	0.68	0.69	0.73	0.73	0.76		
Identity confusion	*M* (*SD*)	3.00 (0.74)	2.69 (0.69)^a^	2.86 (0.65)^b^	3.17 (0.73)^c^	3.05 (0.75)^d^	3.05 (0.74)^d^	*F* (4, 28,809.60) = 130.41***	0.036
	α	0.74	0.67	0.64	0.74	0.75	0.75		
Life satisfaction	*M* (*SD*)	3.49 (1.30)	3.76 (1.32)^a^	3.58 (1.22)^b^	3.62 (1.30)^b^	3.45 (1.27)^c^	3.36 (1.32)^d^	*F* (4, 66,700.46) = 36.65***	0.010
	*α*	0.87	0.85	0.84	0.87	0.87	0.74		

*M* mean; *SD* standard deviation; *α* Cronbach’s α coefficient. Based on the Bonferroni correction, *p* < 0.005 indicates significant difference between groups. Means significantly differ from others if they have different superscripts

***p < 0.001

As reported in Table 2, self–disengagement was positively correlated with identity confusion in early adolescents and negatively correlated with identity synthesis and life satisfaction across all age groups. Self–other recognition was positively correlated with identity synthesis in late adolescents and early and middle emerging adults and with identity confusion in early, middle, and late adolescents and middle emerging adults. Meanwhile, self–other recognition was negatively correlated with identity synthesis in early adolescents and with life satisfaction in early and middle adolescents and early and middle emerging adults. Parental trust was positively correlated with identity synthesis and life satisfaction and negatively correlated with identity confusion for all age groups.

**Table 2:** Bivariate correlations among the study variables

Variables	Age groups	2	3	4	5	6
1. Self–disengagement	Early adolescents	0.56***	−0.66***	−0.25***	0.15***	−0.35***
	Middle adolescents	0.49***	−0.55***	−0.18***	0.02	−0.30***
	Late adolescents	0.48***	−0.57***	−0.25***	0.04	−0.32***
	Early emerging adults	0.51***	−0.54***	−0.17***	0.02	−0.28***
	Middle emerging adults	0.50***	−0.53***	−0.16***	0.00	−0.28***
2. Self–other recognition	Early adolescents	—	−0.42***	−0.06*	0.19***	−0.19***
	Middle adolescents	—	−0.27***	0.04	0.05*	−0.09***
	Late adolescents	—	−0.26***	0.09**	0.10***	−0.04
	Early emerging adults	—	−0.21***	0.09***	0.02	−0.03*
	Middle emerging adults	—	−0.23***	0.13***	0.04**	−0.04**
3. Parental trust	Early adolescents		—	0.28***	−0.24***	0.42***
	Middle adolescents		—	0.26***	−0.20***	0.36***
	Late adolescents		—	0.23***	−0.20***	0.36***
	Early emerging adults		—	0.24***	−0.23***	0.34***
	Middle emerging adults		—	0.23***	−0.21***	0.31***
4. Identity synthesis	Early adolescents			—	−0.33***	0.51***
	Middle adolescents			—	−0.32***	0.45***
	Late adolescents			—	−0.38***	0.55***
	Early emerging adults			—	−0.43***	0.54***
	Middle emerging adults			—	−0.38***	0.54***
5. Identity confusion	Early adolescents				—	−0.41***
	Middle adolescents				—	−0.41***
	Late adolescents				—	−0.37***
	Early emerging adults				—	−0.40***
	Middle emerging adults				—	−0.37***
6. Life satisfaction						—

*p < 0.05, **p < 0.01, ***p < 0.001

### Profiles of Parent–Youth Relationship Quality (Objective 1)

**Fig. 1:**
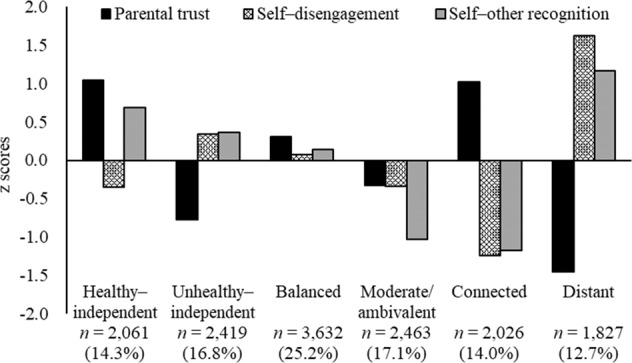
*Z*-scores of parental trust, self–disengagement, and self–other recognition for the six relationship profiles

The two-step cluster analyses indicated that a six-cluster solution is the most acceptable (Fig. 1). These clusters showed theoretically meaningful profiles, as follows. *Healthy–independent* was marked by high parental trust, low self–disengagement, and high self–other recognition. *Unhealthy–independent* was characterized by low parental trust and slightly high self–disengagement and self–other recognition. *Balanced* showed moderate parental trust, self–disengagement, and self–other recognition. *Moderate/ambivalent* constituted slightly low parental trust and self–disengagement and low self–other recognition. *Connected* was marked by high parental trust and low self–disengagement and self–other recognition. *Distant* was characterized by low parental trust and high self–disengagement and self–other recognition. Regarding parsimony, one cluster in the seven-cluster solution was similar to the *moderate/ambivalent* cluster and did not add variation. Further, the six-cluster solution explained 71.3, 60.9, and 64.8% of the variance in self–disengagement, self–other recognition, and parental trust, respectively.

These clusters are consistent with Hypotheses 1a, 1b, and 1c. In line with Hypothesis 1a, the *connected* profile with high parental trust and low emotional separation (both self–disengagement and self–other recognition) was identified. Moreover, consistent with Hypothesis 1b, the *healthy–independent* profile that had both high parental trust and self–other recognition and low self–disengagement was extracted. Further, four interim profiles were identified between *connected* and *healthy–independent*, in line with Hypothesis 1c.

### Age Differences in Profiles of Parent–Youth Relationship Quality (Objective 2)

Distributions of age groups across the six profiles of parent–youth relationship quality were examined by a chi-square test and showed a significant result, χ^2^ (20, *N* = 14,428) = 865.69–920.05, *p*s < 0.001, Cramer’s *V*s = 0.12–0.13, *p*s < 0.001; pooled *F* (20, 3644.41) = 41.94, *p* < 0.001. As shown in Table 3, the adjusted standardized residuals revealed that early adolescents were overrepresented in the *moderate/ambivalent* and *connected* profiles and underrepresented in the *healthy–independent, unhealthy–independent, balanced*, and *distant* profiles. Middle adolescents were more likely to show the *unhealthy–independent* and *balanced* profiles and less likely to show the *moderate/ambivalent* and *distant* profiles. Late adolescents were more likely to show the *healthy–independent* profile and less likely to show the *moderate/ambivalent* and *distant* profiles. Further, early emerging adults were overrepresented in the *healthy–independent* and *distant* profiles, and underrepresented in the *moderate/ambivalent* and *connected* profiles. Middle emerging adults were more likely to display the *healthy–independent*, *balanced*, and *distant* profiles and less likely to exhibit the *moderate/ambivalent* and *connected* profiles.

**Table 3:** Cross tabulation of relationship profiles and age groups

Age groups	Indices	Relationship profiles						Total (%)
		Healthy–independent	Unhealthy–independent	Balanced	Moderate/ambivalent	Connected	Distant	
Early adolescents	Frequency	**5.1 (−)**	**13.1 (−)**	**14.8 (−)**	**28.3 (+)**	**31.1 (+)**	**7.6 (−)**	100
	ASR	**−11.6**	**−4.4**	**−10.7**	**13.3**	**21.8**	**−6.8**	
Middle adolescents	Frequency	13.6	**18.7 (+)**	**29.1 (+)**	**14.8 (−)**	14.3	**9.5 (−)**	100
	ASR	−0.8	**2.3**	**4.1**	**−2.8**	0.3	**−4.3**	
Late adolescents	Frequency	**18.1 (+)**	17.5	24.4	**14.6 (−)**	14.6	**10.8 (−)**	100
	ASR	**4.6**	0.8	−0.7	**−2.8**	0.7	**−2.4**	
Early emerging adults	Frequency	**15.4 (+)**	16.7	26.2	**15.9 (−)**	**10.9 (−)**	**14.9 (+)**	100
	ASR	**2.7**	−0.2	1.9	**−2.6**	**−7.4**	**5.6**	
Middle emerging adults	Frequency	**15.5 (+)**	17.2	**26.8 (+)**	**15.8 (−)**	**10.4 (−)**	**14.2 (+)**	100
	ASR	**2.9**	1.0	**3.1**	**−2.8**	**−8.6**	**3.7**	
Total (%)		14.3	16.8	25.2	17.1	14.0	12.7	100

Observed values indicated in bold are significantly different from expected values (tested by using adjusted residual analysis); (+) indicates that the observed value is higher than the expected value; (−) indicates that the observed value is lower than the expected value

*ASR* adjusted standardized residual

These findings partly support Hypotheses 2a and 2b. In early adolescence, the *connected* profile was the most prevalent, which partially supports Hypothesis 2a (as this was not found in middle adolescence). Moreover, late adolescents and early and middle emerging adults were overrepresented in the *healthy–independent* profile, which is consistent with Hypothesis 2b.

### Links between Profiles of Parent–Youth Relationship Quality and Psychosocial Adjustment (Objective 3)

A series of ANOVAs were performed for identity synthesis, identity confusion, and life satisfaction. Regarding identity synthesis, the main effect of relationship profiles was significant (*F* (5, 8297.22) = 155.21, *p* < 0.001, partial η^2^ = 0.053), whereas the interaction effect of relationship profiles and age groups was non-significant (*F* (20, 1664.02) = 1.34, *p* = 0.141, partial η^2^ = 0.002). Table 4 reports post-hoc analyses by Bonferroni-adjusted tests. Specifically, the *healthy–independent* profile scored the highest on identity synthesis, followed by the *connected*, *balanced*, *moderate/ambivalent*, *unhealthy–independent*, and *distant* profiles.

**Table 4:** The ANOVAs’ post-hoc comparisons for the relationship profile

Variables	Age groups	Relationship profiles						*F*-values	Partial η^2^
		Healthy–independent	Unhealthy–independent	Balanced	Moderate/ambivalent	Connected	Distant		
		*M* (*SD*)	*M* (*SD*)	*M* (*SD*)	*M* (*SD*)	*M* (*SD*)	*M* (*SD*)		
Identity synthesis		3.36 (0.79)^a^	2.88 (0.76)^b^	3.09 (0.72)^c^	2.90 (0.64)^b^	3.24 (0.76)^d^	2.86 (0.91)^b^	*F* (5, 8297.22) = 155.21***	0.053
Identity confusion		2.85 (0.81)^a^	3.20 (0.69)^b^	2.97 (0.70)^c^	3.03 (0.63)^c^	2.79 (0.73)^a^	3.14 (0.82)^b^	*F* (5, 14,600.39) = 108.49***	0.037
Life satisfaction	Early adolescents	4.40 (1.19)^a^	3.04 (1.20)^b^	3.81 (1.18)^c^	3.59 (1.12)^c^	4.31 (1.25)^a^	2.84 (1.55)^b^	*F* (5, 2474.09) = 57.01***	0.152
	Middle adolescents	4.08 (1.25)^a^	3.22 (1.07)^b^	3.65 (1.08)^c^	3.51 (1.16)^c^	4.08 (1.28)^a^	2.78 (1.31)^d^	*F* (5, 1845.94) = 35.81***	0.083
	Late adolescents	4.07 (1.36)^a^	3.23 (1.23)^b^	3.75 (1.08)^c^	3.49 (1.06)^b,c^	4.06 (1.25)^a^	2.81 (1.39)^d^	*F* (5, 12,997.41) = 32.88***	0.096
	Early emerging adults	4.00 (1.31)^a^	3.06 (1.21)^b^	3.61 (1.15)^c^	3.35 (1.08)^d^	3.83 (1.23)^a^	2.84 (1.34)^e^	*F* (5, 9186.54) = 91.80***	0.094
	Middle emerging adults	3.94 (1.39)^a^	3.01 (1.23)^b^	3.40 (1.23)^c^	3.40 (1.04)^c^	3.83 (1.30)^a^	2.68 (1.39)^d^	*F* (5, 8007.45) = 90.85***	0.093

*M* mean; *SD* standard deviation. Based on the Bonferroni correction, *p* < 0.003 indicates significant difference between groups. Means significantly differ from others if they have different superscripts

***p < 0.001

For identity confusion, the main effect of relationship profiles was significant (*F* (5, 14,600.39) = 155.21, *p* < 0.001, partial η^2^ = 0.037), while the interaction effect of relationship profiles and age groups was non-significant (*F* (20, 890.52) = 1.22, *p* = 0.233, partial η^2^ = 0.002). Post-hoc Bonferroni-adjusted tests indicated that the scores of identity confusion were highest in the *unhealthy–independent* and *distant* profiles, followed by the *moderate/ambivalent*, *balanced*, *healthy*–*independent*, and *connected* profiles.

Regarding life satisfaction, both the main effect of relationship profiles (*F* (5, 3907.55) = 310.30, *p* < 0.001, partial η^2^ = 0.103) and interaction effect of relationship profiles and age groups (*F* (20, 9491.90) = 2.13, *p* = 0.002, partial η^2^ = 0.003) were significant. Post-hoc Bonferroni-adjusted tests indicated that the *healthy*–*independent* and *connected* profiles scored the highest on life satisfaction among all age groups. Moreover, the *unhealthy–independent* and *distant* profiles scored the lowest on life satisfaction among early adolescents, whereas the *distant* profile scored the lowest among middle and late adolescents and early and middle emerging adults.

These findings partly support Hypotheses 3a and 3b. Regardless of age, the *healthy–independent* profile scored the highest on identity synthesis, and both the *healthy–independent* and *connected* profiles scored the lowest on identity confusion and highest on life satisfaction.

### Sensitivity Analyses

To address concerns regarding the influences of gender and residential areas, the main analyses (age differences in the distributions of relationship profiles and associations between the relationship profiles and psychosocial adjustment) were conducted, with gender and residential area as the moderators. Except for a few results (e.g., boys/men in the *unhealthy–independent* profile scored higher on identity confusion than boys/men in the *distant* profile) the findings were substantially identical to the original, highlighting the findings’ robustness. Details of these sensitivity analyses are reported in Online Resource 4.

## Discussion

Although separation–individuation and attachment theories indicate how the parent–youth relationship changes and youth become independent from parents, there was a lack of evidence supporting these theoretical notions; a more comprehensive understanding of this process was needed. To address this gap, this study first identified different profiles of parent–youth relationship quality, focusing on emotional separation and parental trust. Then, age differences in the prevalence of young people among the profiles of parent–youth relationship quality were examined. Finally, the associations of parent–youth relationship profiles with psychosocial adjustment were investigated. Overall, the results revealed diverse profiles of parent–youth relationship quality and meaningful age differences in their prevalence and associations with identity and life satisfaction.

### Different Profiles of Parent–Youth Relationship Quality (Objective 1)

Using key components self–disengagement, self–other recognition, and parental trust, this study identified six profiles: *healthy–independent*, *unhealthy–independent*, *balanced*, *moderate/ambivalent*, *connected*, and *distant* relationship. In line with Hypothesis 1a, the *connected* profile with high parental trust and low emotional separation (both self–disengagement and self–other recognition) was extracted. Moreover, consistent with Hypothesis 1b, the *healthy–independent* profile that had both high parental trust and high self–other recognition and low self–disengagement was extracted. These two profiles share high parental trust; however, they differ regarding the level of emotional separation. The *connected* profile is characterized by a strong emotional tie with parents without attaining emotional separation; the *healthy–independent* profile maintains a strong emotional bond with parents and exerts a type of emotional separation, in recognition that the self and parents are separate individuals (self–other recognition) and is low on conflictual separation from parents (self–disengagement). Thus, the latter profile achieves a more nuanced or mature balance between separation and connection.

Four interim profiles were also identified between *connected* and *healthy–independent*, in line with Hypothesis 1c. Two of them—*unhealthy–independent* and *distant*—revealed high separation from parents with low parental trust. The *unhealthy–independent* profile was further characterized by slightly high self–disengagement and self–other recognition, while the *distant* profile was represented by very high self–disengagement and self–other recognition. The former may represent a transitory state of separating from parents, where one struggles with disengaging from infantile parental representation and trying to objectively recognize that they and their parents are different individuals (Beyers et al., 2003); the latter has a weak relationship with their parents. The other two profiles are the *balanced* profile, characterized by moderate scores on all three relational components, and the *moderate/ambivalent* profile, characterized by moderately low scores on the components. Both are moderate types of profiles but are different: the former reveals certain levels of relationships with parents, whereas the latter shows a lack of parental trust but also non-attainment of separation from parents.

Although there is limited research on the profiles of emotional separation and parental trust (e.g., Sugimura et al., 2018), the current findings suggest that there are substantial individual differences in parent–youth relationship quality, from adolescence to emerging adulthood. Thus, not all youth experience a single or an optimal type of balance (e.g., *healthy–independent*) vis-à-vis these relational components in the process of becoming independent from parents, highlighting the importance of acknowledging the multidimensional nature of this process (Allen & Tan, 2016).

### Age Differences in Profiles of Parent–Youth Relationship Quality (Objective 2)

In early adolescence, the *connected* profile was the most prevalent, which partially supports Hypothesis 2a (as this was not found in middle adolescence). Moreover, in late adolescents and early and middle emerging adults, the *healthy–independent* profile were overrepresented, which supports Hypothesis 2b. Additionally, in early and middle emerging adults, the *distant* profile was predominant. This suggests that children enter adolescence with a strong emotional connection with parents and, from late adolescence to emerging adulthood, they disengage from parents in various ways (i.e., at different levels of emotional separation). These findings align with theoretical notions concerning the general trend of changes in balance among key relational components, proposed by Blos’ separation–individuation (Koepke & Denissen, 2012) and Bowlby’s attachment (Allen & Tan, 2016) theories.

It is intriguing that the *unhealthy–independent* profile was prevalent among middle adolescents. This profile is characterized by slightly high self–disengagement and self–other recognition with low parental trust and might suggest the transitory state of emotional separation from parents. Middle adolescents might use emotional separation as a “stepping stone” (Beyers et al., 2003, p. 361) from the connected to a more mature relationship characterized by both emotional separation and parental trust. Moreover, in early and middle emerging adulthood, the *distant* profile was overrepresented, while the *connected* profile was underrepresented. These results suggest that a strong emotional connection with parents is not a pressing issue for youth in older age groups, and a more separated relationship quality is normative (Theisen et al., 2018). This finding aligns with prior attachment studies demonstrating that adolescents’ increased distance from their parents is attributed to their effort to establish an autonomous sense of self and receive more support from peers (Allen et al., 2022) and romantic partners (Oudekerk et al., 2015) as they age. Thus, although secure attachment is essential across the lifespan, older youth struggle with (or enjoy) a more separated relationship with their parents.

These results demonstrate how the balance between emotional separation and parental trust differs from adolescence to emerging adulthood. Using the key components of parental trust and emotional separation, this study provides insights into the process of independence among Japanese adolescents and emerging adults, which is in line with the theoretical notions widely shared across Western cultural contexts (i.e., separation–individuation and attachment theories). As studies in other Asian countries are lacking at present (Sugimura et al., 2021), it is difficult to determine whether the present findings are unique to Japanese youth or overlap with other Asian youth. Therefore, future studies are needed to compare these findings from Japan’s collectivistic culture with other collectivistic Asian countries.

### Age Differences in the Association of Parent–Youth Relationship Quality Profiles with Identity and Life Satisfaction (Objective 3)

The results partly support Hypotheses 3a and 3b, demonstrating that the *healthy–independent* and *connected* profiles scored higher on psychosocial adjustment (high identity synthesis and life satisfaction and low identity confusion) than others in all age groups. Specifically, regarding identity confusion and life satisfaction, both the *healthy–independent* and *connected* profiles revealed the lowest levels of identity confusion and the highest levels of life satisfaction. However, as for identity synthesis, the *healthy–independent* profile scored the highest. These results suggest the significance of connection with parents—represented by a high parental trust—in youth’s psychosocial adjustment (Cooke et al., 2019). However, the results also suggest that identity synthesis requires not only parental trust but also emotional separation (especially self–other recognition). This may imply that, on the one hand, identity develops within a secure attachment context (Kerpelman & Pittman, 2018); on the other hand, it also needs the perception of the self and the parent as separate individuals (represented by self–other recognition). This critical role of self–other recognition may indicate the importance of *distinctiveness* between the self and parents in identity synthesis (van Doeselaar et al., 2019). Individual distinctiveness is the degree of perceived differences between oneself and others in personal characteristics and is believed to be a core factor that makes individuals develop an adoptive identity. Therefore, self–other recognition in parent–youth relationships may benefit identity synthesis.

Moreover, regarding life satisfaction, the *unhealthy–independent* and *distant* profiles obtained the lowest scores in early adolescence, whereas the *distant* profile had the lowest score from middle adolescence to middle emerging adulthood. This suggests that slightly high emotional separation with low parental trust seem less harmful for life satisfaction in older age groups than in early adolescent age group. This aligns with the theoretical notion that as youth age, they seek more distinctive perspectives from their parents (Grotevant & Cooper, 1985) and establish a more separated and mature parent–child relationship (Branje, 2018).

Further, the results have a valuable practical implication that profiles with high parental trust (*healthy–independent*, *balanced*, and *connected*) may promote identity synthesis and life satisfaction and prevent identity confusion, suggesting the importance of a warm, trustful relationship with parents, regardless of age. Youth with profiles characterized by low parental trust and high emotional separation (*unhealthy–independent* and *distant*) are at potential risk of maladjustment. This remarkable finding should be considered when developing support interventions by counselors and practitioners, regardless of the age group. Regulating the relationship between parents and youth and increasing the level of parental trust may be essential for youth psychosocial adjustment (Cooke et al., 2019). Moreover, meeting adolescents’ basic needs for both autonomy and connection contribute to their well-being (Laporte et al., 2021). Thus, maintaining the balance between emotional separation and parental trust is crucial, and counselors and health care practitioners should pay particular attention to those in the *unhealthy–independent* and *distant* profiles.

These results demonstrate that the *healthy–independent* and *connected* profiles are preferable for all age groups, given the higher psychosocial adjustment. In contrast to previous studies using a variable-centered approach (e.g., Sugimura et al., 2018), the present study adopted a person-centered approach, and therefore, the results contribute to identifying specific groups of youth at risk, considering the relationship with their parents (identity confusion in the *unhealthy–independent* and *distant* profiles). Thus, the novelty of this study is that it clarifies the association between parent–youth relationships and psychosocial adjustment through profiles that illustrate concrete problems caused by an imbalance between emotional separation and parental trust.

### Limitations and Future Directions

This study was not without limitations. First, a cross-sectional design was employed. Although several participants with a wide age range (12–25 years) were included, and significant age differences in the prevalence of profiles and in their association with psychosocial adjustment were revealed, it did not allow us to understand how the predominant profiles and the links between profiles and psychosocial adjustment change over time during adolescence and emerging adulthood. Future research should adopt a longitudinal design and study the development of and changes in parent–youth relationships and their links with psychosocial adjustment over time.

Second, all study variables were self-reported. As their cognitive maturation allows young people to recognize their own external and internal situations objectively and accurately, self-reports are the most suitable procedure. Nevertheless, to provide more rigorous evidence, future research should include other measures, such as multi-informant assessments, to corroborate the current findings.

Third, the early, middle, and late adolescence groups mainly comprised participants from urban areas, whereas the early and middle emerging adulthood groups included youth from across Japan. Generally, in East Asia, youth living in urban areas enjoy a more individuated lifestyle than their counterparts in rural areas (Sugimura et al., 2021), and this may have been reflected in the results, especially regarding participants’ relationship with their parents. Furthermore, early and middle adolescence groups had a smaller proportion of female participants. Although this imbalance in residential areas and gender did not have large effects on the findings (see the section on Sensitivity Analyses), future studies should collect gender-balanced data from early and middle adolescents and examine the reproducibility of the present results.

Fourth, this study was conducted in Japan where parent–youth closeness is highly valued, and this characteristic might have affected the results. To unravel how cultural aspects shape the process of becoming independent, future studies should include measures that assess the context (e.g., cultural values and socioeconomic variation).

Fifth, this study did not capture which specific caregiver(s) the youth were representing when they responded to the parent–youth relationship measures. Although this study was able to grasp participants’ general representation of parent(s) or caregiver(s), the quality of their relationships with different caregivers (e.g., fathers and mothers) may affect their process of becoming independent. Therefore, this issue should be considered in future studies.

Finally, the internal consistency of the scales assessing self–other recognition and identity were relatively low, particularly for the adolescent age groups. Although the values were generally equivalent to those reported in previous studies, the low reliability might suggest that self–other recognition and a sense of identity are meaningful to older age groups but less so to younger age groups. Future studies should also clarify how adolescents understand the items.

## Conclusion

Becoming independent from parents is a crucial long-run task for youth and can be characterized by a nuanced balance between emotional separation and parental trust. Yet, the profiles using key components of independence (emotional separation and parental trust) have not been identified, and the age differences in the prevalence of the profiles and in the relationship with psychosocial adjustment were unexplored. This study contributes substantially to the knowledge of diverse profiles of parent–youth relationship quality using fundamental relationship components—emotional separation and parental trust—that constitute the process of becoming independent from parents. Moreover, the novelty of this study is that it provides evidence for the predominant relationship profiles across different age groups, from *connected* profiles (represented by parental trust) to more separated ones (represented by emotional separation). These profiles are meaningfully associated with identity and life satisfaction in youth from early adolescence to middle emerging adulthood. From a person-centered perspective on the dynamics between individual emotional separation and parental trust, the results illuminate the significance of each relational component vis-à-vis identity and life satisfaction. The novelty of this study is that parental trust was fundamental across all age groups, while emotional separation was a necessary component for identity synthesis in all age groups. This positive role of emotional separation has not been found in previous studies, which separately assessed emotional separation and parental trust—emotional separation did not benefit identity development and life satisfaction. Thus, the results can be applied to the development of theory on parent–youth relationships, as well as in intervention programs for psychosocial adjustment from adolescence to emerging adulthood.

### Supplementary information

Supplementary Material

## Appendix

**Psychological Independence Scale**

(1) My opinion sometimes differs from that of my parent.

(2) My lifestyle [my way of living] differs from that of my parent.

(3) My parent and I are separate human beings.

(4) I view my parent as an individual.

(5) I do what’s on my mind instead of what my parent tells me.

*Note*. Response options are *strongly disagree* (1), *disagree* (2), *somewhat disagree, somewhat agree* (3), *agree* (4), and *strongly agree* (5).

## Footnotes

1 As an ancillary analysis, a latent profile analysis (LPA) was conducted to extract the profiles of parent–youth relationship quality. However, it did not identify reliable profiles with good classification accuracy (see more details of the results of LPA in Online Resource 2). Thus, this study reported the results from cluster analyses.
