# Supplementary material for: Profiles of Emotional Separation and Parental Trust from Adolescence to Emerging Adulthood: Age Differences and Associations with Identity and Life Satisfaction
Source: J Youth Adolesc. 2022 Dec 16;52(3):475–89. doi: 10.1007/s10964-022-01716-z (PMC9884255; doi:10.1007/s10964-022-01716-z)
Supplement: Supplementary file 1 — Supplementary Material [file 10964_2022_1716_MOESM1_ESM.docx]

**Supplementary Material**

**Profiles of Emotional Separation and Parental Trust from Adolescence to Emerging Adulthood: Age Differences and Associations with Identity and Life Satisfaction**

Kazumi Sugimura^1^, Shogo Hihara^2^, Kai Hatano^3^, Reiko Nakama^4^, Satoko Saiga^1^, and Manabu Tsuzuki^5^

^1^ Graduate School of Humanities and Social Sciences, Hiroshima University; 1-1-1 Kagamiyama, Higashihiroshima-city, Hiroshima 739-8524, Japan

^2^ Faculty of Business Administration, Matsuyama University, 4-2 Bunkyo-cho, Matsuyama-city, Ehime 790-8578, Japan

^3^ Graduate School of Sustainable System Science, Osaka Metropolitan University; 1-1 Gakuen-cho, Naka-ku, Sakai-city, Osaka 599-8531, Japan

^4^ Graduate School of Education, Hyogo University of Teacher Education; 942-1 Shimokume, Kato-city, Hyogo 673-1494, Japan

^5^ Faculty of Letters, Chuo University; 742-1 Higashinakano, Hachioji-city, Tokyo 192-0393, Japan

**Author Note**

Correspondence concerning this article should be addressed to Kazumi Sugimura, Graduate School of Humanities and Social Sciences, Hiroshima University, 1-1-1 Kagamiyama, Higashihiroshima-city, Hiroshima 739-8524, Japan

Email: ksugimura@hiroshima-u.ac.jp

Phone: +81-82-424-6882

**Index**

**Measurement Invariance Tests…………………………………………….……………….3**

**Latent Profile Analysis for Identifying the Profiles of Parent–Youth Relationship Quality……………………………………….……………………………………………….6**

**Detailed Results Based on the Multiple Imputation Procedure…………...…………….11**

**Sensitivity Analyses…………………………………………………...……………………52**

**Online Resource 1**

**Measurement Invariance Tests**

Measurement invariance of the study variables across age groups was tested by a series of multigroup confirmatory factor analyses (CFAs). Model estimation was conducted using the Full Information Maximum Likelihood estimation (FIML; Enders, 2010) with a maximum likelihood robust strategy (Satorra & Bentler, 2001) in Mplus 8.3 (Muthén & Muthén, 1998–2022). The measurement model included three latent variables for parent–youth relationships (self–disengagement, self–other recognition, and parental trust), two latent variables for identity (synthesis and confusion), and one latent variable for life satisfaction. Furthermore, an item-parceling approach in a random fashion, in which parcels of items load on each latent variable (four parcels or items for each latent variable), was utilized. Specifically, all four parcels of self–disengagement included four items. One parcel of self–other recognition, one parcel of identity synthesis, two parcels of identity confusion, and one parcel of life satisfaction consisted of two items.

The measurement invariance across age groups was tested by three different models: configural (the number of factors and pattern of fixation are equal across groups), metric (the number of factors, pattern of fixation, and factor loadings are equal across groups), and scalar (the number of factors, pattern of fixation, and factor loadings, and item intercepts are equal across groups) invariant models. When the full metric or scalar invariance was not established, ancillary analyses to test partial measurement invariance (some but not all factor loadings or intercepts are equal across groups; Byrne et al., 1989) were conducted. The model fit was evaluated using the comparative fit index (CFI) and the root mean square error of approximation (RMSEA). CFI values higher than .900 indicate an acceptable fit, and RMSEA values less than .080 can be considered acceptable (Byrne, 2012). Differences between model fit were examined based on differences in CFI (ΔCFI) and RMSEA (ΔRMSEA). Values of ΔCFI lower than −.010 supplemented by values of ΔRMSEA higher than .015 indicate the invariance of different models (Chen, 2007).

Table S1 reports the results of measurement invariance tests. Regarding parent–youth relationship, full metric invariance was confirmed, but full scalar invariance was not. Thus, partial scalar invariance was examined by releasing constraints of two parcels for self–disengagement, one parcel and one item for self–other recognition, and two items for parental trust across age groups, according to the modification indices. With these constraints released, partial scalar invariance was established. Regarding identity, full metric invariance was established, but full scalar invariance was rejected. Therefore, partial scalar invariance was explored by freeing constraints of one parcel and one item for identity synthesis and one parcel and two items for identity confusion across age groups. Partial scalar invariance was found with these constraints removed. Nonetheless, identity confusion should be compared with caution because only one of four indicators for identity confusion could be constrained across age groups. As for life satisfaction, full metric invariance was supported, but full scalar invariance could not be established. Partial scalar invariance was confirmed by releasing constraints of one item across age groups.

Table S1

*Tests of Measurement Invariance Across Age Groups*

| Models | Model fit indices | | | |  | Model comparisons | | |
| --- | --- | --- | --- | --- | --- | --- | --- | --- |
|  | χ^2^ | *df* | CFI | RMSEA [90%CI] |  | Pairs | ΔCFI | ΔRMSEA |
| Parent–youth relationship |  |  |  |  |  |  |  |  |
| M1. Configural invariance | 3,321.146 | 255 | .938 | .065 [.063–.067] |  |  |  |  |
| M2. Metric invariance | 3,481.743 | 291 | .935 | .062 [.060–.064] |  | M2−M1 | −.003 | −.003 |
| M3. Full scalar invariance | 5,162.306 | 339 | .902 | .071 [.069–.073] |  | M3−M2 | −.032 | .009 |
| M4. Partial scalar invariance | 4,044.598 | 315 | .925 | .065 [.063–.066] |  | M4−M2 | −.010 | .003 |
| Identity |  |  |  |  |  |  |  |  |
| M1. Configural invariance | 904.104 | 95 | .958 | .055 [.052–.058] |  |  |  |  |
| M2. Metric invariance | 948.988 | 119 | .957 | .050 [.047–.053] |  | M2−M1 | −.001 | −.005 |
| M3. Full scalar invariance | 1,798.315 | 151 | .915 | .062 [.060–.065] |  | M3−M2 | −.043 | .012 |
| M4. Partial scalar invariance | 1,102.472 | 131 | .950 | .051 [.049–.054] |  | M4−M2 | −.008 | .001 |
| Life satisfaction |  |  |  |  |  |  |  |  |
| M1. Configural invariance | 44.208 | 10 | .998 | .035 [.025–.046] |  |  |  |  |
| M2. Metric invariance | 78.279 | 22 | .996 | .030 [.023–.038] |  | M2−M1 | −.002 | −.005 |
| M3. Full scalar invariance | 338.935 | 38 | .981 | .053 [.048–.058] |  | M3−M2 | −.017 | .018 |
| M4. Partial scalar invariance | 197.789 | 34 | .990 | .041 [.036–.047] |  | M4−M2 | −.008 | .006 |

*Note*. M = Model; CFI = comparative ﬁt index; RMSEA = root mean square error of approximation; 90% CI = 90% conﬁdence interval; Δ = change in parameter.

**Online Resource 2**

**Latent Profile Analysis for Identifying the Profiles of Parent–Youth Relationship Quality**

As an ancillary analysis to extract profiles of parent–youth relationship quality, a latent profile analysis (LPA) was performed using Mplus 8.3. One- to seven-profile models were estimated. This study used the following five criteria to define the number of latent profiles. First, sample size adjusted Bayesian information criterion (SSA-BIC; Schwartz, 1978) was utilized. Lower values of SSA-BIC indicate better model fits. Second, the bootstrap Lo-Mendell-Rubin likelihood ratio test (LMR-LRT; Lo et al., 2001) was used. It tests the added value of the *k* profile model over the *k*−1 profile model. A significant LMR-LRT indicates that adding the profile results in a better model fit. Third, the value of entropy, which refers to the degree to which the model-implied classifications correspond to the observed classification, was measured. The values of entropy range from 0 to 1, and values higher than 0.75 suggest accurate classification (Reinecke, 2006). Fourth, for the replicability of profiles, every profile needs to include at least 1% of the entire sample (Hill et al., 2000). Fifth, the interpretability of the extracted profiles was considered (Meeus et al., 2012).

Table S2 and Figure S1 show the results of comparisons among the one- to seven-profiles models. The values of SSA-BIC decreased as the number of profiles increased from one to seven. The results of the LMR-LRT were significant for all models from one to seven profiles. However, except for the two-profile model, the values of entropy were lower than 0.75, which suggested inaccurate classification. The five-, six-, and seven-profile models included at least one profile that comprised lower than 1% of the entire sample. Although these four criteria suggested that only the two-profile model was acceptable, this model extracted no profiles with a high level of parental trust. This result was not considered to capture the diversity of the profiles adequately, and hence, the interpretability of the two-profile model was not supported. Therefore, the LPA could not identify reliable profiles with good classification accuracy.

Table S2

*Fit Indices of a Series of LPA Solutions*

| Profiles | SSA-BIC | Entropy | LMR-LRT |
| --- | --- | --- | --- |
| One profile | 105,839.66 | – | – |
| Two profiles | 100,325.35 | 0.780 | 5,398.100*** |
| Three profiles | 98,525.64 | 0.680 | 1,778.462*** |
| Four profiles | 97,737.57 | 0.672 | 792.690*** |
| Five profiles | 97,263.13 | 0.717 | 487.066*** |
| Six profiles | 96,921.11 | 0.703 | 358.035* |
| Seven profiles | 96,491.34 | 0.658 | 443.529*** |

*Note*. SSA-BIC = sample size adjusted Bayesian information criterion;

LMR-LRT = Lo-Mendell-Rubin likelihood ratio test.

**p* < .05, ****p* < .001.

**Figure S1**

*Z-Scores of Parental Trust, Self–Disengagement, and Self–Other Recognition for Each Model*

**
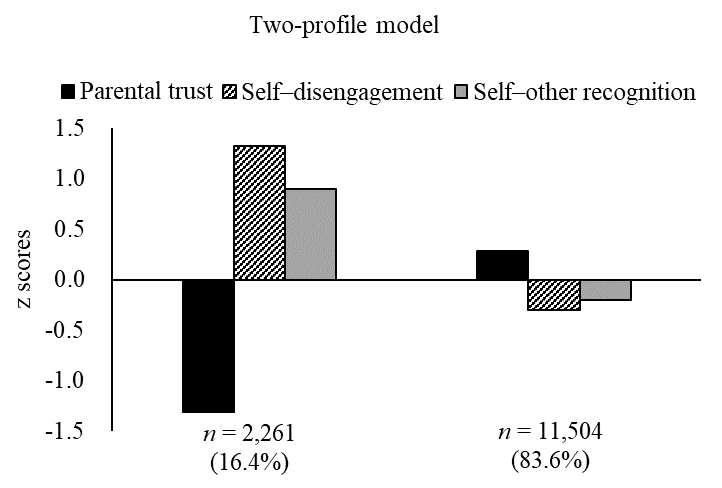

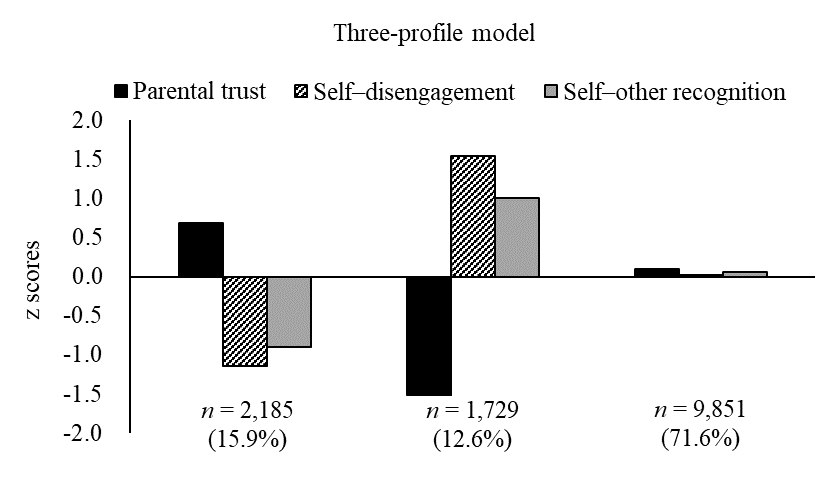
**

**
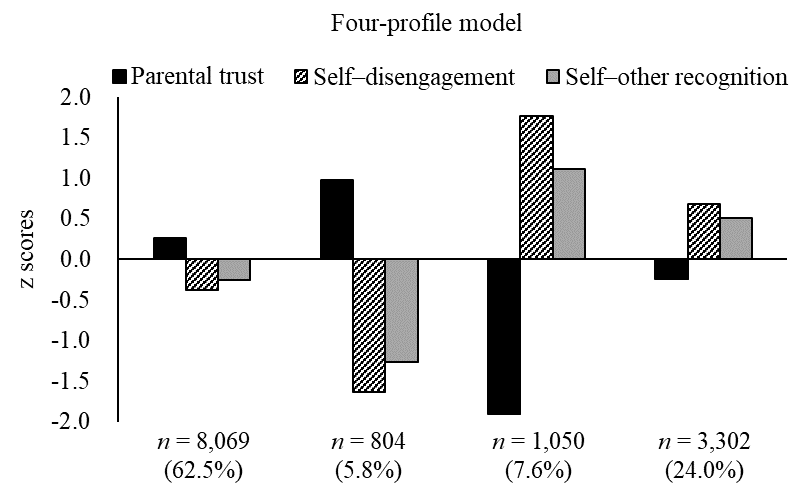

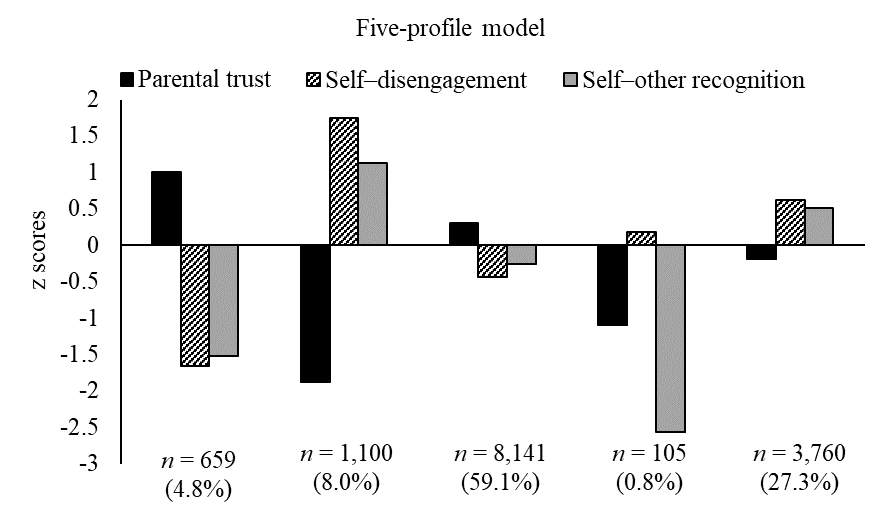
**

**Figure S1**

*(Continued)*

**
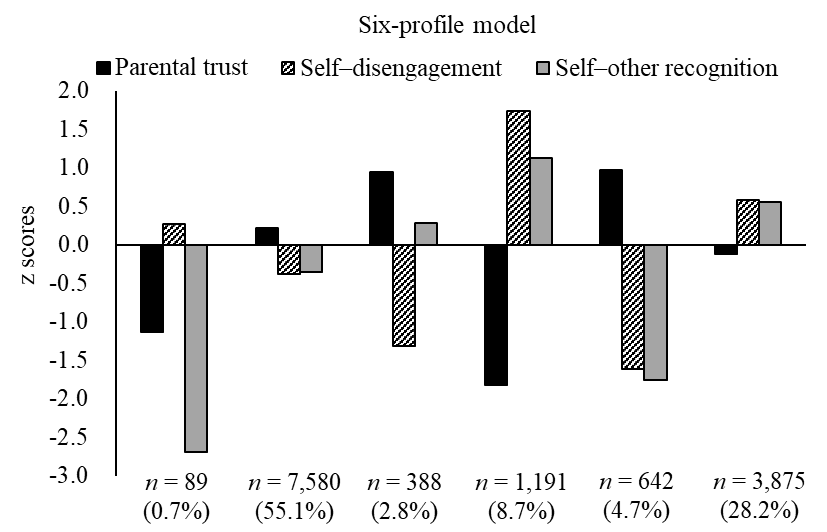

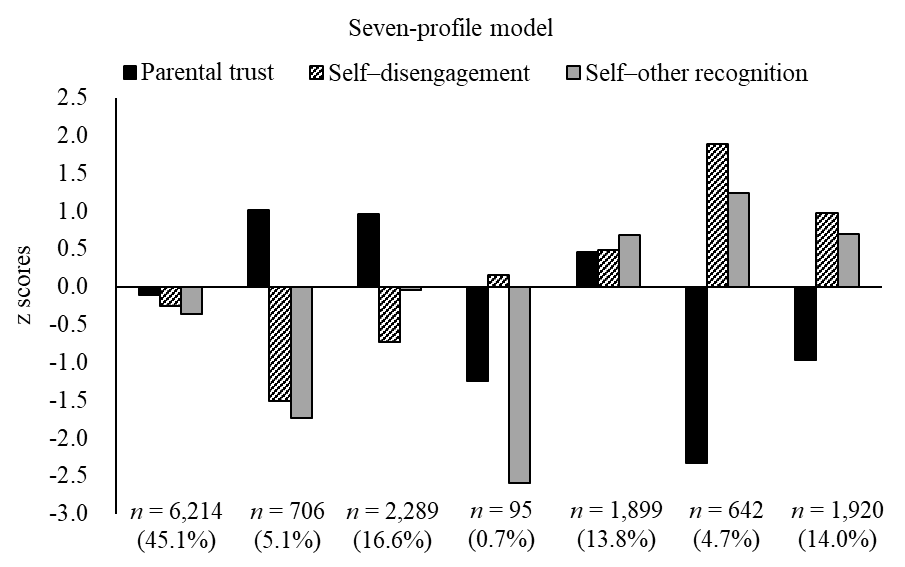
**

**Online Resource 3**

**Detailed Results Based on the Multiple Imputation Procedure**

To deal with missing values, the multiple imputation procedure was performed using SPSS 25.0 (Arbuckle, 2016) and R 4.2.1. This procedure replaced missing values with a 25-iteration setting and produced a total of 20 multiple imputed datasets. Statistical analyses were conducted on each imputed dataset, and the imputed datasets were combined to yield a single set of pooled results. However, for residual analyses following chi-square tests and post-hoc Bonferroni-adjusted tests following ANOVAs, single pooled results cannot be calculated using SPSS or R. Hence, the results of the analyses of all 20 imputed datasets were reported here. For both residual analyses (see Tables S3–S22) and post-hoc Bonferroni-adjusted tests (see Tables S23–S42), the results were substantially consistent across 20 imputed datasets.

Table S3

*Cross-tabulation of Relationship Profiles and Age Groups in the First Dataset*

| Age groups | Indices | Relationship profiles | | | | | | Total (%) |
| --- | --- | --- | --- | --- | --- | --- | --- | --- |
|  |  | Healthy–independent | Unhealthy–independent | Balanced | Moderate/ ambivalent | Connected | Distant |  |
| Early adolescents | Frequency | **5.1 (−)** | **13.1 (−)** | **14.8 (−)** | **28.3 (+)** | **31.1 (+)** | **7.6 (−)** | 100 |
|  | ASR | **−11.6** | **−4.4** | **−10.7** | **13.3** | **21.8** | **−6.8** |  |
|  |  |  |  |  |  |  |  |  |
| Middle adolescents | Frequency | 13.6 | **18.7 (+)** | **29.1 (+)** | **14.8 (−)** | 14.3 | **9.5 (−)** | 100 |
|  | ASR | −0.8 | **2.3** | **4.1** | **−2.8** | 0.3 | **−4.3** |  |
|  |  |  |  |  |  |  |  |  |
| Late adolescents | Frequency | **18.1 (+)** | 17.5 | 24.4 | **14.6 (−)** | 14.6 | **10.8 (−)** | 100 |
|  | ASR | **4.6** | 0.8 | −0.7 | **−2.8** | 0.7 | **−2.4** |  |
|  |  |  |  |  |  |  |  |  |
| Early emerging adults | Frequency | **15.4 (+)** | 16.7 | 26.2 | **15.9 (−)** | **10.9 (−)** | **14.9 (+)** | 100 |
|  | ASR | **2.7** | −0.2 | 1.9 | **−2.6** | **−7.4** | **5.6** |  |
|  |  |  |  |  |  |  |  |  |
| Middle emerging adults | Frequency | **15.5 (+)** | 17.2 | **26.8 (+)** | **15.8 (−)** | **10.4 (−)** | **14.2 (+)** | 100 |
|  | ASR | **2.9** | 1.0 | **3.1** | **−2.8** | **−8.6** | **3.7** |  |
|  |  |  |  |  |  |  |  |  |
| Total (%) |  | 14.3 | 16.8 | 25.2 | 17.1 | 14.0 | 12.7 | 100 |

*Note.* Observed values indicated in bold are significantly different from expected values (tested by using adjusted residual analysis); (+) indicates that the observed value is higher than the expected value; (−) indicates that the observed value is lower than the expected value.

ASR = adjusted standardized residual.

Table S4

*Cross-tabulation of Relationship Profiles and Age Groups in the Second Dataset*

| Age groups | Indices | Relationship profiles | | | | | | Total (%) |
| --- | --- | --- | --- | --- | --- | --- | --- | --- |
|  |  | Healthy–independent | Unhealthy–independent | Balanced | Moderate/ ambivalent | Connected | Distant |  |
| Early adolescents | Frequency | **5.2 (−)** | **13.3 (−)** | **14.5 (−)** | **27.9 (+)** | **31.2 (+)** | **7.8 (−)** | 100 |
|  | ASR | **−11.6** | **−4.0** | **−10.8** | **13.0** | **21.6** | **−6.6** |  |
|  |  |  |  |  |  |  |  |  |
| Middle adolescents | Frequency | 13.8 | **18.9 (+)** | **28.2 (+)** | **14.7 (−)** | 15.0 | **9.5 (−)** | 100 |
|  | ASR | −0.7 | **2.6** | **3.4** | **−2.8** | 1.0 | **−4.5** |  |
|  |  |  |  |  |  |  |  |  |
| Late adolescents | Frequency | **17.8 (+)** | 16.5 | 24.2 | **15.0 (−)** | 15.3 | **11.1 (−)** | 100 |
|  | ASR | **4.3** | −0.2 | −0.7 | **−2.3** | 1.3 | **−2.1** |  |
|  |  |  |  |  |  |  |  |  |
| Early emerging adults | Frequency | **15.4 (+)** | 16.5 | **26.3 (+)** | **15.8 (−)** | **11.0 (−)** | **15.0 (+)** | 100 |
|  | ASR | **2.7** | −0.5 | **2.5** | **−2.6** | **−7.7** | **5.4** |  |
|  |  |  |  |  |  |  |  |  |
| Middle emerging adults | Frequency | **15.6 (+)** | 17.4 | **26.6 (+)** | **15.6 (−)** | **10.5 (−)** | **14.3 (+)** | 100 |
|  | ASR | **3.0** | 1.5 | **3.1** | **−3.0** | **−8.9** | **3.9** |  |
|  |  |  |  |  |  |  |  |  |
| Total (%) |  | 14.3 | 16.7 | 25.0 | 17.0 | 14.2 | 12.8 | 100 |

*Note.* Observed values indicated in bold are significantly different from expected values (tested by using adjusted residual analysis); (+) indicates that the observed value is higher than the expected value; (−) indicates that the observed value is lower than the expected value.

ASR = adjusted standardized residual.

Table S5

*Cross-tabulation of Relationship Profiles and Age Groups in the Third Dataset*

| Age groups | Indices | Relationship profiles | | | | | | Total (%) |
| --- | --- | --- | --- | --- | --- | --- | --- | --- |
|  |  | Healthy–independent | Unhealthy–independent | Balanced | Moderate/ ambivalent | Connected | Distant |  |
| Early adolescents | Frequency | **5.5 (−)** | **12.9 (−)** | **14.1 (−)** | **29.2 (+)** | **30.8 (+)** | **7.6 (−)** | 100 |
|  | ASR | **−11.2** | **−4.5** | **−11.2** | **14.0** | **21.6** | **−6.8** |  |
|  |  |  |  |  |  |  |  |  |
| Middle adolescents | Frequency | 13.8 | **18.9 (+)** | **28.9 (+)** | **14.9 (−)** | 13.8 | **9.7 (−)** | 100 |
|  | ASR | −0.7 | **2.6** | **4.0** | **−2.8** | −0.2 | **−4.2** |  |
|  |  |  |  |  |  |  |  |  |
| Late adolescents | Frequency | **18.2 (+)** | 17.1 | 23.7 | **14.7 (−)** | 15.0 | 11.3 | 100 |
|  | ASR | **4.7** | 0.5 | −1.3 | **−2.9** | 1.3 | −1.8 |  |
|  |  |  |  |  |  |  |  |  |
| Early emerging adults | Frequency | **15.5 (+)** | 16.5 | **26.4 (+)** | **16.0 (−)** | **10.9 (−)** | **14.7 (+)** | 100 |
|  | ASR | **2.7** | −0.3 | **2.6** | **−2.8** | **−7.3** | **4.9** |  |
|  |  |  |  |  |  |  |  |  |
| Middle emerging adults | Frequency | **15.3 (+)** | 17.3 | **26.7 (+)** | **15.9 (−)** | **10.4 (−)** | **14.3 (+)** | 100 |
|  | ASR | **2.4** | 1.3 | **3.3** | **−3.0** | **−8.4** | **4.1** |  |
|  |  |  |  |  |  |  |  |  |
| Total (%) |  | 14.3 | 16.7 | 25.0 | 17.3 | 14.0 | 12.7 | 100 |

*Note.* Observed values indicated in bold are significantly different from expected values (tested by using adjusted residual analysis); (+) indicates that the observed value is higher than the expected value; (−) indicates that the observed value is lower than the expected value.

ASR = adjusted standardized residual.

Table S6

*Cross-tabulation of Relationship Profiles and Age Groups in the Fourth Dataset*

| Age groups | Indices | Relationship profiles | | | | | | Total (%) |
| --- | --- | --- | --- | --- | --- | --- | --- | --- |
|  |  | Healthy–independent | Unhealthy–independent | Balanced | Moderate/ ambivalent | Connected | Distant |  |
| Early adolescents | Frequency | **5.3 (−)** | **13.4 (−)** | **14.7 (−)** | **28.0 (+)** | **30.8 (+)** | **7.8 (−)** | 100 |
|  | ASR | **−11.5** | **−4.0** | **−10.5** | **12.8** | **21.5** | **−6.7** |  |
|  |  |  |  |  |  |  |  |  |
| Middle adolescents | Frequency | 14.0 | **18.9 (+)** | **28.7 (+)** | **14.6 (−)** | 14.4 | **9.5 (−)** | 100 |
|  | ASR | −0.5 | **2.5** | **3.9** | **−3.0** | 0.4 | **−4.5** |  |
|  |  |  |  |  |  |  |  |  |
| Late adolescents | Frequency | **18.1 (+)** | 16.8 | 23.9 | **15.0 (−)** | 15.0 | **11.1 (−)** | 100 |
|  | ASR | **4.5** | 0.1 | −1.0 | **−2.3** | 1.2 | **−2.2** |  |
|  |  |  |  |  |  |  |  |  |
| Early emerging adults | Frequency | **15.5 (+)** | 16.6 | **26.2 (+)** | **16.0 (−)** | **10.7 (−)** | **15.0 (+)** | 100 |
|  | ASR | **2.7** | −0.4 | **2.4** | **−2.4** | **−7.9** | **5.4** |  |
|  |  |  |  |  |  |  |  |  |
| Middle emerging adults | Frequency | **15.5 (+)** | 17.4 | **26.4 (+)** | **15.8 (−)** | **10.6 (−)** | **14.3 (+)** | 100 |
|  | ASR | **2.7** | 1.4 | **2.8** | **−2.9** | **−8.2** | **3.9** |  |
|  |  |  |  |  |  |  |  |  |
| Total (%) |  | 14.4 | 16.8 | 24.9 | 17.1 | 14.0 | 12.8 | 100 |

*Note.* Observed values indicated in bold are significantly different from expected values (tested by using adjusted residual analysis); (+) indicates that the observed value is higher than the expected value; (−) indicates that the observed value is lower than the expected value.

ASR = adjusted standardized residual.

Table S7

*Cross-tabulation of Relationship Profiles and Age Groups in the Fifth Dataset*

| Age groups | Indices | Relationship profiles | | | | | | Total (%) |
| --- | --- | --- | --- | --- | --- | --- | --- | --- |
|  |  | Healthy–independent | Unhealthy–independent | Balanced | Moderate/ ambivalent | Connected | Distant |  |
| Early adolescents | Frequency | **5.4 (−)** | **13.2 (−)** | **14.0 (−)** | **28.4 (+)** | **31.3 (+)** | **7.7 (−)** | 100 |
|  | ASR | **−11.4** | **−4.0** | **−11.2** | **13.3** | **21.9** | **−6.8** |  |
|  |  |  |  |  |  |  |  |  |
| Middle adolescents | Frequency | 14.5 | **18.5 (+)** | **28.0 (+)** | **15.1 (−)** | 14.2 | **9.7 (−)** | 100 |
|  | ASR | 0.2 | **2.4** | **3.3** | **−2.5** | 0.1 | **−4.3** |  |
|  |  |  |  |  |  |  |  |  |
| Late adolescents | Frequency | **18.0 (+)** | 16.9 | 23.6 | **15.2 (−)** | 14.9 | 11.4 | 100 |
|  | ASR | **4.4** | 0.4 | −1.3 | **−2.2** | 0.9 | −1.9 |  |
|  |  |  |  |  |  |  |  |  |
| Early emerging adults | Frequency | **15.3 (+)** | 16.5 | **26.3 (+)** | **15.9 (−)** | **10.9 (−)** | **15.1 (+)** | 100 |
|  | ASR | **2.2** | −0.2 | **2.7** | **−2.8** | **−7.7** | **5.6** |  |
|  |  |  |  |  |  |  |  |  |
| Middle emerging adults | Frequency | **15.5 (+)** | 17.1 | **26.8 (+)** | **15.7 (−)** | **10.7 (−)** | **14.3 (+)** | 100 |
|  | ASR | **2.7** | 1.1 | **3.6** | **−3.2** | **−8.2** | **3.5** |  |
|  |  |  |  |  |  |  |  |  |
| Total (%) |  | 14.4 | 16.6 | 24.9 | 17.2 | 14.2 | 12.8 | 100 |

*Note.* Observed values indicated in bold are significantly different from expected values (tested by using adjusted residual analysis); (+) indicates that the observed value is higher than the expected value; (−) indicates that the observed value is lower than the expected value.

ASR = adjusted standardized residual.

Table S8

*Cross-tabulation of Relationship Profiles and Age Groups in the Sixth Dataset*

| Age groups | Indices | Relationship profiles | | | | | | Total (%) |
| --- | --- | --- | --- | --- | --- | --- | --- | --- |
|  |  | Healthy–independent | Unhealthy–independent | Balanced | Moderate/ ambivalent | Connected | Distant |  |
| Early adolescents | Frequency | **5.3 (−)** | **13.0 (−)** | **14.7 (−)** | **28.1 (+)** | **31.1 (+)** | **7.8 (−)** | 100 |
|  | ASR | **−11.5** | **−4.4** | **−10.6** | **13.0** | **21.7** | **−6.6** |  |
|  |  |  |  |  |  |  |  |  |
| Middle adolescents | Frequency | 13.6 | **18.6 (+)** | **28.5 (+)** | **14.6 (−)** | 14.9 | **9.7 (−)** | 100 |
|  | ASR | −0.9 | **2.4** | **3.7** | **−3.0** | 1.0 | **−4.2** |  |
|  |  |  |  |  |  |  |  |  |
| Late adolescents | Frequency | **18.2 (+)** | 17.0 | 23.7 | **14.7 (−)** | 15.2 | **11.2 (−)** | 100 |
|  | ASR | **4.7** | 0.4 | −1.3 | **−2.6** | 1.3 | **−2.0** |  |
|  |  |  |  |  |  |  |  |  |
| Early emerging adults | Frequency | **15.4 (+)** | 16.6 | **26.5 (+)** | **15.9 (−)** | **10.8 (−)** | **14.8 (+)** | 100 |
|  | ASR | **2.6** | −0.1 | **2.8** | **−2.7** | **−7.9** | **4.9** |  |
|  |  |  |  |  |  |  |  |  |
| Middle emerging adults | Frequency | **15.6 (+)** | 17.2 | **26.5 (+)** | **15.9 (−)** | **10.4 (−)** | **14.4 (+)** | 100 |
|  | ASR | **2.9** | 1.2 | **2.8** | **−2.5** | **−8.8** | **4.0** |  |
|  |  |  |  |  |  |  |  |  |
| Total (%) |  | 14.3 | 16.7 | 25.0 | 17.1 | 14.1 | 12.8 | 100 |

*Note.* Observed values indicated in bold are significantly different from expected values (tested by using adjusted residual analysis); (+) indicates that the observed value is higher than the expected value; (−) indicates that the observed value is lower than the expected value.

ASR = adjusted standardized residual.

Table S9

*Cross-tabulation of Relationship Profiles and Age Groups in the Seventh Dataset*

| Age groups | Indices | Relationship profiles | | | | | | Total (%) |
| --- | --- | --- | --- | --- | --- | --- | --- | --- |
|  |  | Healthy–independent | Unhealthy–independent | Balanced | Moderate/ ambivalent | Connected | Distant |  |
| Early adolescents | Frequency | **5.4 (−)** | **12.8 (−)** | **14.5 (−)** | **28.2 (+)** | **31.3 (+)** | **7.8 (−)** | 100 |
|  | ASR | **−11.3** | **−4.7** | **−10.7** | **13.3** | **21.9** | **−6.8** |  |
|  |  |  |  |  |  |  |  |  |
| Middle adolescents | Frequency | 13.9 | **18.7 (+)** | **28.4 (+)** | **14.6 (−)** | 14.3 | **10.1 (−)** | 100 |
|  | ASR | −0.6 | **2.4** | **3.7** | **−2.9** | 0.1 | **−3.7** |  |
|  |  |  |  |  |  |  |  |  |
| Late adolescents | Frequency | **18.0 (+)** | 17.1 | 23.5 | **14.9 (−)** | 15.2 | **11.3 (−)** | 100 |
|  | ASR | **4.5** | 0.4 | −1.3 | **−2.4** | 1.2 | **−2.0** |  |
|  |  |  |  |  |  |  |  |  |
| Early emerging adults | Frequency | **15.4 (+)** | 16.8 | **26.2 (+)** | **15.8 (−)** | **10.8 (−)** | **14.9 (+)** | 100 |
|  | ASR | **2.7** | 0.2 | **2.5** | **−2.7** | **−7.9** | **4.9** |  |
|  |  |  |  |  |  |  |  |  |
| Middle emerging adults | Frequency | **15.4 (+)** | 17.2 | **26.6 (+)** | **15.7 (−)** | **10.6 (−)** | **14.5 (+)** | 100 |
|  | ASR | **2.6** | 1.1 | **3.2** | **−2.9** | **−8.3** | **3.8** |  |
|  |  |  |  |  |  |  |  |  |
| Total (%) |  | 14.3 | 16.7 | 24.9 | 17.0 | 14.2 | 12.9 | 100 |

*Note.* Observed values indicated in bold are significantly different from expected values (tested by using adjusted residual analysis); (+) indicates that the observed value is higher than the expected value; (−) indicates that the observed value is lower than the expected value.

ASR = adjusted standardized residual.

Table S10

*Cross-tabulation of Relationship Profiles and Age Groups in the Eighth Dataset*

| Age groups | Indices | Relationship profiles | | | | | | Total (%) |
| --- | --- | --- | --- | --- | --- | --- | --- | --- |
|  |  | Healthy–independent | Unhealthy–independent | Balanced | Moderate/ ambivalent | Connected | Distant |  |
| Early adolescents | Frequency | **5.3 (−)** | **12.8 (−)** | **14.7 (−)** | **28.5 (+)** | **31.1 (+)** | **7.6 (−)** | 100 |
|  | ASR | **−11.5** | **−4.7** | **−10.4** | **13.4** | **21.6** | **−6.8** |  |
|  |  |  |  |  |  |  |  |  |
| Middle adolescents | Frequency | 14.1 | **19.2 (+)** | **28.2 (+)** | **15.1 (−)** | 14.3 | **9.0 (−)** | 100 |
|  | ASR | −0.3 | **3.0** | **3.5** | **−2.5** | 0.2 | **−5.0** |  |
|  |  |  |  |  |  |  |  |  |
| Late adolescents | Frequency | **18.0 (+)** | 17.0 | 23.9 | **14.6 (−)** | 15.4 | **11.1 (−)** | 100 |
|  | ASR | **4.4** | 0.3 | −0.9 | **−2.9** | 1.5 | **−2.0** |  |
|  |  |  |  |  |  |  |  |  |
| Early emerging adults | Frequency | **15.6 (+)** | 16.5 | **26.1 (+)** | **16.0 (−)** | **10.7 (−)** | **15.1 (+)** | 100 |
|  | ASR | **2.7** | −0.6 | **2.3** | **−2.5** | **−8.1** | **6.0** |  |
|  |  |  |  |  |  |  |  |  |
| Middle emerging adults | Frequency | **15.5 (+)** | 17.5 | **26.5 (+)** | **15.7 (−)** | **10.7 (−)** | **14.2 (+)** | 100 |
|  | ASR | **2.6** | 1.6 | **3.1** | **−3.1** | **−8.1** | **3.6** |  |
|  |  |  |  |  |  |  |  |  |
| Total (%) |  | 14.4 | 16.7 | 24.9 | 17.1 | 14.1 | 12.7 | 100 |

*Note.* Observed values indicated in bold are significantly different from expected values (tested by using adjusted residual analysis); (+) indicates that the observed value is higher than the expected value; (−) indicates that the observed value is lower than the expected value.

ASR = adjusted standardized residual.

Table S11

*Cross-tabulation of Relationship Profiles and Age Groups in the Nineth Dataset*

| Age groups | Indices | Relationship profiles | | | | | | Total (%) |
| --- | --- | --- | --- | --- | --- | --- | --- | --- |
|  |  | Healthy–independent | Unhealthy–independent | Balanced | Moderate/ ambivalent | Connected | Distant |  |
| Early adolescents | Frequency | **5.7 (−)** | **12.9 (−)** | **14.4 (−)** | **27.9 (+)** | **31.3 (+)** | **7.8 (−)** | 100 |
|  | ASR | **−11.1** | **−4.4** | **−10.9** | **12.8** | **22.2** | **−6.7** |  |
|  |  |  |  |  |  |  |  |  |
| Middle adolescents | Frequency | 14.5 | **18.4 (+)** | **28.3 (+)** | **14.7 (−)** | 14.0 | **10.1 (−)** | 100 |
|  | ASR | 0.1 | **2.2** | **3.4** | **−2.9** | 0.0 | **−3.6** |  |
|  |  |  |  |  |  |  |  |  |
| Late adolescents | Frequency | **18.2 (+)** | 17.5 | 23.9 | **14.8 (−)** | 14.8 | **10.8 (−)** | 100 |
|  | ASR | **4.5** | 1.1 | −1.2 | **−2.6** | 1.0 | **−2.5** |  |
|  |  |  |  |  |  |  |  |  |
| Early emerging adults | Frequency | **15.6 (+)** | 16.2 | **26.6 (+)** | **16.0 (−)** | **10.7 (−)** | **14.9 (+)** | 100 |
|  | ASR | **2.6** | −0.8 | **2.9** | **−2.4** | **−7.9** | **5.3** |  |
|  |  |  |  |  |  |  |  |  |
| Middle emerging adults | Frequency | **15.4 (+)** | 17.3 | **26.7 (+)** | **15.8 (−)** | **10.6 (−)** | **14.2 (+)** | 100 |
|  | ASR | **2.1** | 1.6 | **3.1** | **−2.8** | **−8.2** | **3.6** |  |
|  |  |  |  |  |  |  |  |  |
| Total (%) |  | 14.5 | 16.6 | 25.1 | 17.1 | 14.0 | 12.8 | 100 |

*Note.* Observed values indicated in bold are significantly different from expected values (tested by using adjusted residual analysis); (+) indicates that the observed value is higher than the expected value; (−) indicates that the observed value is lower than the expected value.

ASR = adjusted standardized residual.

Table S12

*Cross-tabulation of Relationship Profiles and Age Groups in the Tenth Dataset*

| Age groups | Indices | Relationship profiles | | | | | | Total (%) |
| --- | --- | --- | --- | --- | --- | --- | --- | --- |
|  |  | Healthy–independent | Unhealthy–independent | Balanced | Moderate/ ambivalent | Connected | Distant |  |
| Early adolescents | Frequency | **5.3 (−)** | **13.3 (−)** | **13.8 (−)** | **28.5 (+)** | **31.3 (+)** | **7.8 (−)** | 100 |
|  | ASR | **−11.4** | **−4.0** | **−11.3** | **13.4** | **21.9** | **−6.8** |  |
|  |  |  |  |  |  |  |  |  |
| Middle adolescents | Frequency | 14.4 | **18.4 (+)** | **28.4 (+)** | **14.5 (−)** | 14.3 | **10.1 (−)** | 100 |
|  | ASR | 0.0 | **2.1** | **3.8** | **−3.2** | 0.1 | **−3.7** |  |
|  |  |  |  |  |  |  |  |  |
| Late adolescents | Frequency | **18.1 (+)** | 17.0 | 23.9 | **14.7 (−)** | 15.4 | **10.9 (−)** | 100 |
|  | ASR | **4.6** | 0.4 | −0.9 | **−2.8** | 1.5 | **−2.5** |  |
|  |  |  |  |  |  |  |  |  |
| Early emerging adults | Frequency | **15.3 (+)** | 16.6 | **26.3 (+)** | **15.9 (−)** | **10.9 (−)** | **15.0 (+)** | 100 |
|  | ASR | **2.3** | 0.0 | **2.8** | **−2.8** | **−7.8** | **5.2** |  |
|  |  |  |  |  |  |  |  |  |
| Middle emerging adults | Frequency | **15.4 (+)** | 17.1 | **26.4 (+)** | **16.0 (−)** | **10.6 (−)** | **14.4 (+)** | 100 |
|  | ASR | **2.5** | 1.0 | **3.1** | **−2.4** | **−8.5** | **3.8** |  |
|  |  |  |  |  |  |  |  |  |
| Total (%) |  | 14.3 | 16.6 | 24.8 | 17.1 | 14.2 | 12.9 | 100 |

*Note.* Observed values indicated in bold are significantly different from expected values (tested by using adjusted residual analysis); (+) indicates that the observed value is higher than the expected value; (−) indicates that the observed value is lower than the expected value.

ASR = adjusted standardized residual.

Table S13

*Cross-tabulation of Relationship Profiles and Age Groups in the Eleventh Dataset*

| Age groups | Indices | Relationship profiles | | | | | | Total (%) |
| --- | --- | --- | --- | --- | --- | --- | --- | --- |
|  |  | Healthy–independent | Unhealthy–independent | Balanced | Moderate/ ambivalent | Connected | Distant |  |
| Early adolescents | Frequency | **5.6 (−)** | **13.6 (−)** | **14.5 (−)** | **27.7 (+)** | **31.0 (+)** | **7.6 (−)** | 100 |
|  | ASR | **−11.3** | **−3.6** | **−10.7** | **12.6** | **21.8** | **−6.9** |  |
|  |  |  |  |  |  |  |  |  |
| Middle adolescents | Frequency | 14.1 | 17.8 | **29.1 (+)** | **14.9 (−)** | 14.1 | **10.0 (−)** | 100 |
|  | ASR | −0.5 | 1.4 | **4.3** | **−2.5** | 0.2 | **−3.8** |  |
|  |  |  |  |  |  |  |  |  |
| Late adolescents | Frequency | **18.2 (+)** | 17.0 | 24.2 | **14.5 (−)** | 15.0 | **11.0 (−)** | 100 |
|  | ASR | **4.5** | 0.4 | −0.7 | **−2.8** | 1.2 | **−2.3** |  |
|  |  |  |  |  |  |  |  |  |
| Early emerging adults | Frequency | **15.5 (+)** | 16.6 | **26.2 (+)** | **15.9 (−)** | **10.8 (−)** | **15.0 (+)** | 100 |
|  | ASR | **2.4** | −0.1 | **2.3** | **−2.5** | **−7.6** | **5.4** |  |
|  |  |  |  |  |  |  |  |  |
| Middle emerging adults | Frequency | **15.7 (+)** | 17.3 | **26.4 (+)** | **15.8 (−)** | **10.5 (−)** | **14.3 (+)** | 100 |
|  | ASR | **2.8** | 1.4 | **2.7** | **−2.6** | **−8.5** | **3.7** |  |
|  |  |  |  |  |  |  |  |  |
| Total (%) |  | 14.5 | 16.7 | 25.0 | 17.0 | 14.0 | 12.8 | 100 |

*Note.* Observed values indicated in bold are significantly different from expected values (tested by using adjusted residual analysis); (+) indicates that the observed value is higher than the expected value; (−) indicates that the observed value is lower than the expected value.

ASR = adjusted standardized residual.

Table S14

*Cross-tabulation of Relationship Profiles and Age Groups in the Twelfth Dataset*

| Age groups | Indices | Relationship profiles | | | | | | Total (%) |
| --- | --- | --- | --- | --- | --- | --- | --- | --- |
|  |  | Healthy–independent | Unhealthy–independent | Balanced | Moderate/ ambivalent | Connected | Distant |  |
| Early adolescents | Frequency | **5.8 (−)** | **13.2 (−)** | **14.1 (−)** | **28.1 (+)** | **31.3 (+)** | **7.6 (−)** | 100 |
|  | ASR | **−11.0** | **−4.2** | **−11.2** | **13.0** | **22.1** | **−6.9** |  |
|  |  |  |  |  |  |  |  |  |
| Middle adolescents | Frequency | 14.0 | **18.9 (+)** | **28.4 (+)** | **14.7 (−)** | 14.4 | **9.7 (−)** | 100 |
|  | ASR | −0.5 | **2.6** | **3.5** | **−2.9** | 0.4 | **−4.2** |  |
|  |  |  |  |  |  |  |  |  |
| Late adolescents | Frequency | **17.8 (+)** | 17.1 | 23.8 | **15.0 (−)** | 15.0 | **11.2 (−)** | 100 |
|  | ASR | **4.2** | 0.4 | −1.2 | **−2.3** | 1.2 | **−2.0** |  |
|  |  |  |  |  |  |  |  |  |
| Early emerging adults | Frequency | **15.4 (+)** | 16.4 | **26.7 (+)** | **16.0 (−)** | **10.6 (−)** | **15.0 (+)** | 100 |
|  | ASR | **2.4** | −0.7 | **3.1** | **−2.5** | **−8.2** | **5.5** |  |
|  |  |  |  |  |  |  |  |  |
| Middle emerging adults | Frequency | **15.6 (+)** | 17.4 | **26.6 (+)** | **15.7 (−)** | **10.5 (−)** | **14.2 (+)** | 100 |
|  | ASR | **2.7** | 1.5 | **3.0** | **−3.0** | **−8.4** | **3.6** |  |
|  |  |  |  |  |  |  |  |  |
| Total (%) |  | 14.4 | 16.7 | 25.0 | 17.1 | 14.0 | 12.7 | 100 |

*Note.* Observed values indicated in bold are significantly different from expected values (tested by using adjusted residual analysis); (+) indicates that the observed value is higher than the expected value; (−) indicates that the observed value is lower than the expected value.

ASR = adjusted standardized residual.

Table S15

*Cross-tabulation of Relationship Profiles and Age Groups in the Thirteenth Dataset*

| Age groups | Indices | Relationship profiles | | | | | | Total (%) |
| --- | --- | --- | --- | --- | --- | --- | --- | --- |
|  |  | Healthy–independent | Unhealthy–independent | Balanced | Moderate/ ambivalent | Connected | Distant |  |
| Early adolescents | Frequency | **5.2 (−)** | **12.9 (−)** | **14.5 (−)** | **28.8 (+)** | **30.9 (+)** | **7.8 (−)** | 100 |
|  | ASR | **−11.6** | **−4.4** | **−10.7** | **13.5** | **21.4** | **−6.6** |  |
|  |  |  |  |  |  |  |  |  |
| Middle adolescents | Frequency | 14.0 | **18.7 (+)** | **28.0 (+)** | **15.5 (−)** | 14.4 | **9.4 (−)** | 100 |
|  | ASR | −0.4 | **2.6** | **3.2** | **−2.1** | 0.3 | **−4.5** |  |
|  |  |  |  |  |  |  |  |  |
| Late adolescents | Frequency | **18.3 (+)** | 16.7 | 24.1 | **15.0 (−)** | 15.0 | **10.9 (−)** | 100 |
|  | ASR | **4.8** | 0.1 | −0.8 | **−2.6** | 1.1 | **−2.4** |  |
|  |  |  |  |  |  |  |  |  |
| Early emerging adults | Frequency | **15.3 (+)** | 16.4 | **26.2 (+)** | **16.0 (−)** | **11.1 (−)** | **15.0 (+)** | 100 |
|  | ASR | **2.4** | −0.4 | **2.4** | **−2.8** | **−7.2** | **5.5** |  |
|  |  |  |  |  |  |  |  |  |
| Middle emerging adults | Frequency | **15.5 (+)** | 17.3 | **26.7 (+)** | **15.7 (−)** | **10.5 (−)** | **14.4 (+)** | 100 |
|  | ASR | **2.7** | 1.6 | **3.3** | **−3.4** | **−8.7** | **3.9** |  |
|  |  |  |  |  |  |  |  |  |
| Total (%) |  | 14.3 | 16.6 | 24.9 | 17.3 | 14.1 | 12.8 | 100 |

*Note.* Observed values indicated in bold are significantly different from expected values (tested by using adjusted residual analysis); (+) indicates that the observed value is higher than the expected value; (−) indicates that the observed value is lower than the expected value.

ASR = adjusted standardized residual.

Table S16

*Cross-tabulation of Relationship Profiles and Age Groups in the Fourteenth Dataset*

| Age groups | Indices | Relationship profiles | | | | | | Total (%) |
| --- | --- | --- | --- | --- | --- | --- | --- | --- |
|  |  | Healthy–independent | Unhealthy–independent | Balanced | Moderate/ ambivalent | Connected | Distant |  |
| Early adolescents | Frequency | **5.2 (−)** | **12.9 (−)** | **14.5 (−)** | **28.8 (+)** | **30.9 (+)** | **7.8 (−)** | 100 |
|  | ASR | **−11.6** | **−4.4** | **−10.7** | **13.5** | **21.4** | **−6.6** |  |
|  |  |  |  |  |  |  |  |  |
| Middle adolescents | Frequency | 14.0 | **18.7 (+)** | **28.0 (+)** | **15.5 (−)** | 14.4 | **9.4 (−)** | 100 |
|  | ASR | −0.4 | **2.6** | **3.2** | **−2.1** | 0.3 | **−4.5** |  |
|  |  |  |  |  |  |  |  |  |
| Late adolescents | Frequency | **18.3 (+)** | 16.7 | 24.1 | **15.0 (−)** | 15.0 | **10.9 (−)** | 100 |
|  | ASR | **4.8** | 0.1 | −0.8 | **−2.6** | 1.1 | **−2.4** |  |
|  |  |  |  |  |  |  |  |  |
| Early emerging adults | Frequency | **15.3 (+)** | 16.4 | **26.2 (+)** | **16.0 (−)** | **11.1 (−)** | **15.0 (+)** | 100 |
|  | ASR | **2.4** | −0.4 | **2.4** | **−2.8** | **−7.2** | **5.5** |  |
|  |  |  |  |  |  |  |  |  |
| Middle emerging adults | Frequency | **15.5 (+)** | 17.3 | **26.7 (+)** | **15.7 (−)** | **10.5 (−)** | **14.4 (+)** | 100 |
|  | ASR | **2.7** | 1.6 | **3.3** | **−3.4** | **−8.7** | **3.9** |  |
|  |  |  |  |  |  |  |  |  |
| Total (%) |  | 14.3 | 16.6 | 24.9 | 17.3 | 14.1 | 12.8 | 100 |

*Note.* Observed values indicated in bold are significantly different from expected values (tested by using adjusted residual analysis); (+) indicates that the observed value is higher than the expected value; (−) indicates that the observed value is lower than the expected value.

ASR = adjusted standardized residual.

Table S17

*Cross-tabulation of Relationship Profiles and Age Groups in the Fifteenth Dataset*

| Age groups | Indices | Relationship profiles | | | | | | Total (%) |
| --- | --- | --- | --- | --- | --- | --- | --- | --- |
|  |  | Healthy–independent | Unhealthy–independent | Balanced | Moderate/ ambivalent | Connected | Distant |  |
| Early adolescents | Frequency | **5.6 (−)** | **13.5 (−)** | **14.6 (−)** | **27.9 (+)** | **31.1 (+)** | **7.4 (−)** | 100 |
|  | ASR | **−11.1** | **−4.0** | **−10.7** | **12.9** | **21.7** | **−7.1** |  |
|  |  |  |  |  |  |  |  |  |
| Middle adolescents | Frequency | 13.6 | **18.6 (+)** | **29.1 (+)** | **14.8 (−)** | 14.5 | **9.5 (−)** | 100 |
|  | ASR | −1.0 | **2.2** | **4.2** | **−2.7** | 0.5 | **−4.3** |  |
|  |  |  |  |  |  |  |  |  |
| Late adolescents | Frequency | **18.3 (+)** | 17.2 | 23.5 | **15.0 (−)** | 15.0 | **11.1 (−)** | 100 |
|  | ASR | **4.8** | 0.4 | −1.5 | **−2.3** | 1.1 | **−2.1** |  |
|  |  |  |  |  |  |  |  |  |
| Early emerging adults | Frequency | **15.3 (+)** | 16.7 | **26.5 (+)** | **15.8 (−)** | **10.9 (−)** | **14.8 (+)** | 100 |
|  | ASR | **2.2** | −0.3 | **2.9** | **−2.6** | **−7.7** | **5.3** |  |
|  |  |  |  |  |  |  |  |  |
| Middle emerging adults | Frequency | **15.6 (+)** | 17.4 | **26.4 (+)** | **15.7 (−)** | **10.5 (−)** | **14.3 (+)** | 100 |
|  | ASR | **3.0** | 1.3 | **2.6** | **−2.9** | **−8.4** | **4.1** |  |
|  |  |  |  |  |  |  |  |  |
| Total (%) |  | 14.4 | 16.8 | 25.0 | 17.0 | 14.1 | 12.7 | 100 |

*Note.* Observed values indicated in bold are significantly different from expected values (tested by using adjusted residual analysis); (+) indicates that the observed value is higher than the expected value; (−) indicates that the observed value is lower than the expected value.

ASR = adjusted standardized residual.

Table S18

*Cross-tabulation of Relationship Profiles and Age Groups in the Sixteenth Dataset*

| Age groups | Indices | Relationship profiles | | | | | | Total (%) |
| --- | --- | --- | --- | --- | --- | --- | --- | --- |
|  |  | Healthy–independent | Unhealthy–independent | Balanced | Moderate/ ambivalent | Connected | Distant |  |
| Early adolescents | Frequency | **5.5 (−)** | **13.4 (−)** | **14.5 (−)** | **27.8 (+)** | **31.2 (+)** | **7.7 (−)** | 100 |
|  | ASR | **−11.2** | **−4.0** | **−10.8** | **12.9** | **21.7** | **−6.8** |  |
|  |  |  |  |  |  |  |  |  |
| Middle adolescents | Frequency | 13.9 | 18.1 | **29.5 (+)** | **14.3 (−)** | 14.4 | **9.8 (−)** | 100 |
|  | ASR | −0.6 | 1.7 | **4.7** | **−3.1** | 0.3 | **−4.1** |  |
|  |  |  |  |  |  |  |  |  |
| Late adolescents | Frequency | **18.3 (+)** | 17.4 | 23.9 | **14.7 (−)** | 14.6 | **11.1 (−)** | 100 |
|  | ASR | **4.8** | 0.7 | −1.2 | **−2.5** | 0.6 | **−2.2** |  |
|  |  |  |  |  |  |  |  |  |
| Early emerging adults | Frequency | **15.3 (+)** | 16.7 | **26.2 (+)** | **15.8 (−)** | **11.0 (−)** | **15.0 (+)** | 100 |
|  | ASR | **2.3** | −0.1 | **2.3** | **−2.5** | **−7.4** | **5.3** |  |
|  |  |  |  |  |  |  |  |  |
| Middle emerging adults | Frequency | **15.4 (+)** | 17.3 | **26.5 (+)** | **15.7 (−)** | **10.7 (−)** | **14.3 (+)** | 100 |
|  | ASR | **2.7** | 1.3 | **2.8** | **−2.6** | **−8.3** | **3.8** |  |
|  |  |  |  |  |  |  |  |  |
| Total (%) |  | 14.3 | 16.8 | 25.0 | 16.9 | 14.1 | 12.8 | 100 |

*Note.* Observed values indicated in bold are significantly different from expected values (tested by using adjusted residual analysis); (+) indicates that the observed value is higher than the expected value; (−) indicates that the observed value is lower than the expected value.

ASR = adjusted standardized residual.

Table S19

*Cross-tabulation of Relationship Profiles and Age Groups in the Seventeenth Dataset*

| Age groups | Indices | Relationship profiles | | | | | | Total (%) |
| --- | --- | --- | --- | --- | --- | --- | --- | --- |
|  |  | Healthy–independent | Unhealthy–independent | Balanced | Moderate/ ambivalent | Connected | Distant |  |
| Early adolescents | Frequency | **5.6 (−)** | **12.9 (−)** | **14.7 (−)** | **28.1 (+)** | **30.9 (+)** | **7.8 (−)** | 100 |
|  | ASR | **−11.1** | **−4.4** | **−10.5** | **13.1** | **21.4** | **−6.8** |  |
|  |  |  |  |  |  |  |  |  |
| Middle adolescents | Frequency | 13.9 | **18.7 (+)** | **28.3 (+)** | **14.7 (−)** | 14.4 | **9.9 (−)** | 100 |
|  | ASR | −0.5 | **2.5** | **3.5** | **−2.8** | 0.4 | **−4.1** |  |
|  |  |  |  |  |  |  |  |  |
| Late adolescents | Frequency | **17.8 (+)** | 16.5 | 24.4 | **14.9 (−)** | 15.2 | **11.2 (−)** | 100 |
|  | ASR | **4.2** | −0.1 | −0.6 | **−2.3** | 1.4 | **−2.2** |  |
|  |  |  |  |  |  |  |  |  |
| Early emerging adults | Frequency | **15.5 (+)** | 16.7 | **26.1 (+)** | **15.8 (−)** | **10.9 (−)** | **15.1 (+)** | 100 |
|  | ASR | **2.8** | 0.1 | **2.1** | **−2.7** | **−7.7** | **5.2** |  |
|  |  |  |  |  |  |  |  |  |
| Middle emerging adults | Frequency | **15.4 (+)** | 17.3 | **26.7 (+)** | **15.6 (−)** | **10.6 (−)** | **14.5 (+)** | 100 |
|  | ASR | **2.5** | 1.3 | **3.2** | **−2.9** | **−8.4** | **3.8** |  |
|  |  |  |  |  |  |  |  |  |
| Total (%) |  | 14.3 | 16.7 | 25.0 | 17.0 | 14.1 | 12.9 | 100 |

*Note.* Observed values indicated in bold are significantly different from expected values (tested by using adjusted residual analysis); (+) indicates that the observed value is higher than the expected value; (−) indicates that the observed value is lower than the expected value.

ASR = adjusted standardized residual.

Table S20

*Cross-tabulation of Relationship Profiles and Age Groups in the Eighteenth Dataset*

| Age groups | Indices | Relationship profiles | | | | | | Total (%) |
| --- | --- | --- | --- | --- | --- | --- | --- | --- |
|  |  | Healthy–independent | Unhealthy–independent | Balanced | Moderate/ ambivalent | Connected | Distant |  |
| Early adolescents | Frequency | **5.9 (−)** | **13.4 (−)** | **14.3 (−)** | **27.9 (+)** | **30.8 (+)** | **7.6 (−)** | 100 |
|  | ASR | **−10.8** | **−4.1** | **−10.9** | **13.0** | **21.4** | **−6.8** |  |
|  |  |  |  |  |  |  |  |  |
| Middle adolescents | Frequency | 13.9 | **19.1 (+)** | **28.3 (+)** | **14.8 (−)** | 14.5 | **9.5 (−)** | 100 |
|  | ASR | −0.7 | **2.8** | **3.5** | **−2.7** | 0.5 | **−4.4** |  |
|  |  |  |  |  |  |  |  |  |
| Late adolescents | Frequency | **18.0 (+)** | 17.1 | 24.1 | **14.9 (−)** | 14.9 | **11.1 (−)** | 100 |
|  | ASR | **4.2** | 0.3 | −0.9 | **−2.3** | 0.9 | **−2.0** |  |
|  |  |  |  |  |  |  |  |  |
| Early emerging adults | Frequency | 15.3 | 16.5 | **26.6 (+)** | **15.6 (−)** | **11.1 (−)** | **15.0 (+)** | 100 |
|  | ASR | 1.9 | −0.6 | **3.1** | **−3.1** | **−7.3** | **5.7** |  |
|  |  |  |  |  |  |  |  |  |
| Middle emerging adults | Frequency | **15.9 (+)** | 17.4 | **26.3 (+)** | **15.8 (−)** | **10.5 (−)** | **14.1 (+)** | 100 |
|  | ASR | **3.3** | 1.3 | **2.6** | **−2.5** | **−8.6** | **3.5** |  |
|  |  |  |  |  |  |  |  |  |
| Total (%) |  | 14.5 | 16.8 | 25.0 | 17.0 | 14.1 | 12.7 | 100 |

*Note.* Observed values indicated in bold are significantly different from expected values (tested by using adjusted residual analysis); (+) indicates that the observed value is higher than the expected value; (−) indicates that the observed value is lower than the expected value.

ASR = adjusted standardized residual.

Table S21

*Cross-tabulation of Relationship Profiles and Age Groups in the Nineteenth Dataset*

| Age groups | Indices | Relationship profiles | | | | | | Total (%) |
| --- | --- | --- | --- | --- | --- | --- | --- | --- |
|  |  | Healthy–independent | Unhealthy–independent | Balanced | Moderate/ ambivalent | Connected | Distant |  |
| Early adolescents | Frequency | **5.1 (−)** | **13.5 (−)** | **14.8 (−)** | **28.0 (+)** | **31.2 (+)** | **7.5 (−)** | 100 |
|  | ASR | **−11.7** | **−4.1** | **−10.7** | **13.1** | **21.8** | **−6.9** |  |
|  |  |  |  |  |  |  |  |  |
| Middle adolescents | Frequency | 14.0 | **18.6 (+)** | **28.8 (+)** | **14.8 (−)** | 14.4 | **9.4 (−)** | 100 |
|  | ASR | −0.3 | **2.1** | **3.8** | **−2.6** | 0.4 | **−4.5** |  |
|  |  |  |  |  |  |  |  |  |
| Late adolescents | Frequency | **17.8 (+)** | 16.8 | 24.4 | **14.8 (−)** | 15.1 | 11.2 | 100 |
|  | ASR | **4.3** | −0.1 | −0.8 | **−2.4** | 1.2 | −1.9 |  |
|  |  |  |  |  |  |  |  |  |
| Early emerging adults | Frequency | **15.4 (+)** | 16.8 | **26.4 (+)** | **15.7 (−)** | **10.8 (−)** | **14.9 (+)** | 100 |
|  | ASR | **2.6** | −0.1 | **2.4** | **−2.7** | **−7.8** | **5.5** |  |
|  |  |  |  |  |  |  |  |  |
| Middle emerging adults | Frequency | **15.5 (+)** | 17.6 | **26.7 (+)** | **15.6 (−)** | **10.5 (−)** | **14.2 (+)** | 100 |
|  | ASR | **2.9** | 1.6 | **2.9** | **−3.0** | **−8.5** | **3.7** |  |
|  |  |  |  |  |  |  |  |  |
| Total (%) |  | 14.3 | 16.9 | 25.2 | 16.9 | 14.1 | 12.7 | 100 |

*Note.* Observed values indicated in bold are significantly different from expected values (tested by using adjusted residual analysis); (+) indicates that the observed value is higher than the expected value; (−) indicates that the observed value is lower than the expected value.

ASR = adjusted standardized residual.

Table S22

*Cross-tabulation of Relationship Profiles and Age Groups in the Twentieth Dataset*

| Age groups | Indices | Relationship profiles | | | | | | Total (%) |
| --- | --- | --- | --- | --- | --- | --- | --- | --- |
|  |  | Healthy–independent | Unhealthy–independent | Balanced | Moderate/ ambivalent | Connected | Distant |  |
| Early adolescents | Frequency | **5.2 (−)** | **13.3 (−)** | **14.7 (−)** | **27.8 (+)** | **31.2 (+)** | **7.8 (−)** | 100 |
|  | ASR | **−11.6** | **−4.0** | **−10.5** | **12.6** | **21.8** | **−6.7** |  |
|  |  |  |  |  |  |  |  |  |
| Middle adolescents | Frequency | 14.3 | **18.5 (+)** | **28.9 (+)** | **15.0 (−)** | 14.1 | **9.2 (−)** | 100 |
|  | ASR | −0.2 | **2.3** | **4.1** | **−2.6** | 0.0 | **−4.9** |  |
|  |  |  |  |  |  |  |  |  |
| Late adolescents | Frequency | **17.9 (+)** | 17.2 | 24.0 | **14.7 (−)** | 15.2 | **11.0 (−)** | 100 |
|  | ASR | **4.3** | 0.6 | −0.9 | **−2.7** | 1.3 | **−2.3** |  |
|  |  |  |  |  |  |  |  |  |
| Early emerging adults | Frequency | **15.3 (+)** | 16.5 | **26.0 (+)** | **16.1 (−)** | **10.9 (−)** | **15.2 (+)** | 100 |
|  | ASR | **2.1** | −0.3 | **2.1** | **−2.2** | **−7.8** | **6.0** |  |
|  |  |  |  |  |  |  |  |  |
| Middle emerging adults | Frequency | **15.7 (+)** | 17.1 | **26.5 (+)** | **15.8 (−)** | **10.6 (−)** | **14.2 (+)** | 100 |
|  | ASR | **3.2** | 1.0 | **2.9** | **−2.9** | **−8.3** | **3.6** |  |
|  |  |  |  |  |  |  |  |  |
| Total (%) |  | 14.4 | 16.7 | 24.9 | 17.1 | 14.1 | 12.8 | 100 |

*Note.* Observed values indicated in bold are significantly different from expected values (tested by using adjusted residual analysis); (+) indicates that the observed value is higher than the expected value; (−) indicates that the observed value is lower than the expected value.

ASR = adjusted standardized residual.

Table S23

*The ANOVAs’ Post-hoc Comparisons for the Relationship Profiles in the First Dataset*

| Variables | Age groups | Relationship profiles | | | | | |
| --- | --- | --- | --- | --- | --- | --- | --- |
|  |  | Healthy–independent | Unhealthy–independent | Balanced | Moderate/ ambivalent | Connected | Distant |
|  |  | *M* (*SD*) | *M* (*SD*) | *M* (*SD*) | *M* (*SD*) | *M* (*SD*) | *M* (*SD*) |
| Identity synthesis |  | 3.36 (0.79)^a^ | 2.88 (0.76)^b^ | 3.09 (0.72)^c^ | 2.90 (0.64)^b^ | 3.24 (0.76)^d^ | 2.86 (0.91)^b^ |
| Identity confusion |  | 2.85 (0.81)^a^ | 3.20 (0.69)^b^ | 2.97 (0.70)^c^ | 3.03 (0.63)^c^ | 2.79 (0.73)^a^ | 3.14 (0.82)^b^ |
| Life satisfaction | Early adolescents | 4.40 (1.19)^a^ | 3.04 (1.20)^b^ | 3.81 (1.18)^c^ | 3.59 (1.12)^c^ | 4.31 (1.25)^a^ | 2.84 (1.55)^b^ |
|  | Middle adolescents | 4.08 (1.25)^a^ | 3.22 (1.07)^b^ | 3.65 (1.08)^c^ | 3.51 (1.16)^c^ | 4.08 (1.28)^a^ | 2.78 (1.31)^d^ |
|  | Late adolescents | 4.07 (1.36)^a^ | 3.23 (1.23)^b^ | 3.75 (1.08)^c^ | 3.49 (1.06)^bc^ | 4.06 (1.25)^a^ | 2.81 (1.39)^d^ |
|  | Early emerging adults | 4.00 (1.31)^a^ | 3.06 (1.21)^b^ | 3.61 (1.15)^c^ | 3.35 (1.08)^d^ | 3.83 (1.23)^a^ | 2.84 (1.34)^e^ |
|  | Middle emerging adults | 3.94 (1.39)^a^ | 3.01 (1.23)^b^ | 3.40 (1.23)^c^ | 3.40 (1.04)^c^ | 3.83 (1.30)^a^ | 2.68 (1.39)^d^ |

*Note. M* = mean; *SD* = standard deviation Based on the Bonferroni correction, *p* < .003 indicates significant difference between groups. Means significantly differ from others if they have different superscripts.

Table S24

*The ANOVAs’ Post-hoc Comparisons for the Relationship Profiles in the Second Dataset*

| Variables | Age groups | Relationship profiles | | | | | |
| --- | --- | --- | --- | --- | --- | --- | --- |
|  |  | Healthy–independent | Unhealthy–independent | Balanced | Moderate/ ambivalent | Connected | Distant |
|  |  | *M* (*SD*) | *M* (*SD*) | *M* (*SD*) | *M* (*SD*) | *M* (*SD*) | *M* (*SD*) |
| Identity synthesis |  | 3.36 (0.79)^a^ | 2.89 (0.76)^b^ | 3.09 (0.73)^c^ | 2.90 (0.64)^b^ | 3.25 (0.76)^d^ | 2.86 (0.91)^b^ |
| Identity confusion |  | 2.84 (0.82)^a^ | 3.19 (0.69)^b^ | 2.97 (0.70)^c^ | 3.02 (0.62)^c^ | 2.79 (0.73)^a^ | 3.16 (0.82)^b^ |
| Life satisfaction | Early adolescents | 4.45 (1.16)^a^ | 3.06 (1.17)^b^ | 3.83 (1.18)^c^ | 3.56 (1.11)^c^ | 4.30 (1.28)^a^ | 2.85 (1.56)^b^ |
|  | Middle adolescents | 4.03 (1.22)^a^ | 3.23 (1.05)^b^ | 3.60 (1.09)^c^ | 3.49 (1.16)^bc^ | 4.10 (1.25)^a^ | 2.84 (1.31)^d^ |
|  | Late adolescents | 4.06 (1.38)^a^ | 3.25 (1.23)^b^ | 3.73 (1.18)^c^ | 3.48 (1.05)^bc^ | 4.07 (1.23)^a^ | 2.81 (1.37)^d^ |
|  | Early emerging adults | 4.00 (1.31)^a^ | 3.06 (1.20)^b^ | 3.61 (1.17)^c^ | 3.38 (1.08)^d^ | 3.83 (1.22)^a^ | 2.85 (1.33)^e^ |
|  | Middle emerging adults | 3.94 (1.39)^a^ | 3.00 (1.23)^b^ | 3.42 (1.22)^c^ | 3.41 (1.03)^c^ | 3.81 (1.28)^a^ | 2.69 (1.40)^d^ |

*Note. M* = mean; *SD* = standard deviation. Based on the Bonferroni correction, *p* < .003 indicates significant difference between groups. Means significantly differ from others if they have different superscripts.

Table S25

*The ANOVAs’ Post-hoc Comparisons for the Relationship Profiles in the Third Dataset*

| Variables | Age groups | Relationship profiles | | | | | |
| --- | --- | --- | --- | --- | --- | --- | --- |
|  |  | Healthy–independent | Unhealthy–independent | Balanced | Moderate/ ambivalent | Connected | Distant |
|  |  | *M* (*SD*) | *M* (*SD*) | *M* (*SD*) | *M* (*SD*) | *M* (*SD*) | *M* (*SD*) |
| Identity synthesis |  | 3.36 (0.78)^a^ | 2.89 (0.76)^b^ | 3.10 (0.72)^c^ | 2.90 (0.64)^b^ | 3.24 (0.76)^d^ | 2.85 (0.92)^b^ |
| Identity confusion |  | 2.84 (0.81)^a^ | 3.20 (0.69)^b^ | 2.97 (0.70)^c^ | 3.02 (0.63)^d^ | 2.79 (0.73)^a^ | 3.16 (0.82)^b^ |
| Life satisfaction | Early adolescents | 4.43 (1.23)^a^ | 3.11 (1.20)^b^ | 3.79 (1.20)^c^ | 3.57 (1.09)^c^ | 4.32 (1.26)^a^ | 2.73 (1.51)^b^ |
|  | Middle adolescents | 4.03 (1.23)^a^ | 3.15 (1.10)^b^ | 3.67 (1.06)^c^ | 3.53 (1.14)^c^ | 4.04 (1.28)^a^ | 2.83 (1.34)^d^ |
|  | Late adolescents | 4.05 (1.39)^a^ | 3.26 (1.22)^b^ | 3.78 (1.16)^a^ | 3.44 (1.03)^b^ | 4.06 (1.24)^a^ | 2.82 (1.38)^c^ |
|  | Early emerging adults | 4.00 (1.31)^a^ | 3.06 (1.19)^b^ | 3.61 (1.15)^c^ | 3.35 (1.07)^d^ | 3.86 (1.21)^a^ | 2.84 (1.35)^e^ |
|  | Middle emerging adults | 3.96 (1.39)^a^ | 3.00 (1.24)^b^ | 3.41 (1.21)^c^ | 3.40 (1.05)^c^ | 3.82 (1.28)^a^ | 2.69 (1.40)^d^ |

*Note. M* = mean; *SD* = standard deviation. Based on the Bonferroni correction, *p* < .003 indicates significant difference between groups. Means significantly differ from others if they have different superscripts.

Table S26

*The ANOVAs’ Post-hoc Comparisons for the Relationship Profiles in the Fourth Dataset*

| Variables | Age groups | Relationship profiles | | | | | |
| --- | --- | --- | --- | --- | --- | --- | --- |
|  |  | Healthy–independent | Unhealthy–independent | Balanced | Moderate/ ambivalent | Connected | Distant |
|  |  | *M* (*SD*) | *M* (*SD*) | *M* (*SD*) | *M* (*SD*) | *M* (*SD*) | *M* (*SD*) |
| Identity synthesis |  | 3.36 (0.78)^a^ | 2.89 (0.76)^b^ | 3.10 (0.72)^c^ | 2.90 (0.64)^b^ | 3.25 (0.76)^d^ | 2.86 (0.91)^b^ |
| Identity confusion |  | 2.84 (0.81)^a^ | 3.21 (0.69)^b^ | 2.97 (0.70)^c^ | 3.02 (0.62)^c^ | 2.78 (0.73)^a^ | 3.15 (0.83)^b^ |
| Life satisfaction | Early adolescents | 4.41 (1.24)^a^ | 3.05 (1.21)^b^ | 3.84 (1.18)^c^ | 3.56 (1.10)^d^ | 4.32 (1.25)^a^ | 2.87 (1.57)^b^ |
|  | Middle adolescents | 4.00 (1.23)^a^ | 3.19 (1.10)^b^ | 3.63 (1.08)^c^ | 3.49 (1.11)^c^ | 4.07 (1.27)^a^ | 2.88 (1.34)^b^ |
|  | Late adolescents | 4.07 (1.40)^a^ | 3.21 (1.21)^b^ | 3.75 (1.16)^c^ | 3.42 (1.04)^b^ | 4.08 (1.25)^a^ | 2.84 (1.41)^d^ |
|  | Early emerging adults | 3.99 (1.29)^a^ | 3.06 (1.21)^b^ | 3.61 (1.15)^c^ | 3.37 (1.07)^d^ | 3.85 (1.22)^a^ | 2.84 (1.35)^e^ |
|  | Middle emerging adults | 3.96 (1.39)^a^ | 2.99 (1.22)^b^ | 3.41 (1.20)^c^ | 3.40 (1.06)^c^ | 3.79 (1.29)^a^ | 2.69 (1.40)^d^ |

*Note. M* = mean; *SD* = standard deviation. Based on the Bonferroni correction, *p* < .003 indicates significant difference between groups. Means significantly differ from others if they have different superscripts.

Table S27

*The ANOVAs’ Post-hoc Comparisons for the Relationship Profiles in the Fifth Dataset*

| Variables | Age groups | Relationship profiles | | | | | |
| --- | --- | --- | --- | --- | --- | --- | --- |
|  |  | Healthy–independent | Unhealthy–independent | Balanced | Moderate/ ambivalent | Connected | Distant |
|  |  | *M* (*SD*) | *M* (*SD*) | *M* (*SD*) | *M* (*SD*) | *M* (*SD*) | *M* (*SD*) |
| Identity synthesis |  | 3.37 (0.79)^a^ | 2.88 (0.75)^b^ | 3.10 (0.72)^c^ | 2.90 (0.64)^b^ | 3.24 (0.76)^d^ | 2.86 (0.91)^b^ |
| Identity confusion |  | 2.85 (0.82)^a^ | 3.20 (0.69)^b^ | 2.97 (0.70)^c^ | 3.02 (0.63)^c^ | 2.79 (0.72)^a^ | 3.16 (0.82)^b^ |
| Life satisfaction | Early adolescents | 4.41 (1.20)^a^ | 3.03 (1.20)^b^ | 3.83 (1.22)^c^ | 3.58 (1.08)^c^ | 4.30 (1.26)^a^ | 2.85 (1.58)^b^ |
|  | Middle adolescents | 4.02 (1.21)^a^ | 3.22 (1.06)^b^ | 3.64 (1.10)^c^ | 3.55 (1.08)^c^ | 4.07 (1.27)^a^ | 2.80 (1.25)^d^ |
|  | Late adolescents | 4.07 (1.39)^a^ | 3.24 (1.21)^b^ | 3.74 (1.19)^c^ | 3.47 (1.05)^bc^ | 4.05 (1.25)^a^ | 2.84 (1.39)^d^ |
|  | Early emerging adults | 3.99 (1.31)^a^ | 3.07 (1.21)^b^ | 3.61 (1.15)^c^ | 3.37 (1.07)^d^ | 3.87 (1.22)^a^ | 2.84 (1.34)^e^ |
|  | Middle emerging adults | 3.96 (1.38)^a^ | 2.98 (1.24)^b^ | 3.40 (1.21)^c^ | 3.40 (1.03)^c^ | 3.82 (1.28)^a^ | 2.71 (1.40)^d^ |

*Note. M* = mean; *SD* = standard deviation. Based on the Bonferroni correction, *p* < .003 indicates significant difference between groups. Means significantly differ from others if they have different superscripts.

Table S28

*The ANOVAs’ Post-hoc Comparisons for the Relationship Profiles in the Sixth Dataset*

| Variables | Age groups | Relationship profiles | | | | | |  |
| --- | --- | --- | --- | --- | --- | --- | --- | --- |
|  |  | Healthy–independent | Unhealthy–independent | Balanced | Moderate/ ambivalent | Connected | Distant | |
|  |  | *M* (*SD*) | *M* (*SD*) | *M* (*SD*) | *M* (*SD*) | *M* (*SD*) | *M* (*SD*) | |
| Identity synthesis |  | 3.36 (0.79)^a^ | 2.89 (0.76)^b^ | 3.10 (0.72)^c^ | 2.90 (0.64)^b^ | 3.24 (0.76)^d^ | 2.86 (0.90)^b^ | |
| Identity confusion |  | 2.84 (0.81)^a^ | 3.19 (0.68)^b^ | 2.97 (0.70)^c^ | 3.03 (0.63)^c^ | 2.78 (0.74)^a^ | 3.15 (0.82)^b^ | |
| Life satisfaction | Early adolescents | 4.42 (1.21)^a^ | 3.06 (1.21)^b^ | 3.78 (1.17)^c^ | 3.58 (1.09)^c^ | 4.31 (1.26)^a^ | 2.84 (1.58)^b^ | |
|  | Middle adolescents | 4.06 (1.19)^a^ | 3.21 (1.06)^b^ | 3.65 (1.08)^c^ | 3.50 (1.14)^c^ | 4.02 (1.28)^a^ | 2.84 (1.31)^d^ | |
|  | Late adolescents | 4.05 (1.41)^a^ | 3.26 (1.20)^b^ | 3.75 (1.20)^cd^ | 3.48 (1.02)^bc^ | 4.03 (1.25)^ad^ | 2.82 (1.38)^e^ | |
|  | Early emerging adults | 3.99 (1.31)^a^ | 3.06 (1.21)^b^ | 3.61 (1.16)^c^ | 3.37 (1.08)^d^ | 3.84 (1.21)^a^ | 2.83 (1.32)^e^ | |
|  | Middle emerging adults | 3.97 (1.37)^a^ | 3.01 (1.23)^b^ | 3.41 (1.23)^c^ | 3.40 (1.04)^c^ | 3.78 (1.31)^a^ | 2.69 (1.40)^d^ | |

*Note. M* = mean; *SD* = standard deviation. Based on the Bonferroni correction, *p* < .003 indicates significant difference between groups. Means significantly differ from others if they have different superscripts.

Table S29

*The ANOVAs’ Post-hoc Comparisons for the Relationship Profiles in the Seventh Dataset*

| Variables | Age groups | Relationship profiles | | | | | |
| --- | --- | --- | --- | --- | --- | --- | --- |
|  |  | Healthy–independent | Unhealthy–independent | Balanced | Moderate/ ambivalent | Connected | Distant |
|  |  | *M* (*SD*) | *M* (*SD*) | *M* (*SD*) | *M* (*SD*) | *M* (*SD*) | *M* (*SD*) |
| Identity synthesis |  | 3.37 (0.79)^a^ | 2.88 (0.76)^b^ | 3.10 (0.72)^c^ | 2.90 (0.64)^b^ | 3.23 (0.76)^d^ | 2.86 (0.91)^b^ |
| Identity confusion |  | 2.84 (0.81)^a^ | 3.21 (0.68)^b^ | 2.97 (0.70)^c^ | 3.02 (0.63)^c^ | 2.79 (0.73)^a^ | 3.15 (0.82)^b^ |
| Life satisfaction | Early adolescents | 4.42 (1.20)^a^ | 3.00 (1.17)^b^ | 3.79 (1.20)^c^ | 3.59 (1.12)^c^ | 4.31 (1.24)^a^ | 2.82 (1.58)^b^ |
|  | Middle adolescents | 4.08 (1.23)^a^ | 3.22 (1.05)^b^ | 3.62 (1.08)^c^ | 3.50 (1.15)^c^ | 4.03 (1.27)^a^ | 2.87 (1.32)^d^ |
|  | Late adolescents | 4.05 (1.41)^a^ | 3.27 (1.23)^b^ | 3.73 (1.17)^c^ | 3.47 (1.05)^bc^ | 4.08 (1.22)^a^ | 2.84 (1.38)^d^ |
|  | Early emerging adults | 4.02 (1.31)^a^ | 3.05 (1.21)^b^ | 3.62 (1.15)^c^ | 3.38 (1.06)^d^ | 3.85 (1.23)^a^ | 2.84 (1.33)^e^ |
|  | Middle emerging adults | 3.96 (1.40)^a^ | 2.98 (1.21)^b^ | 3.43 (1.21)^c^ | 3.40 (1.06)^c^ | 3.81 (1.29)^a^ | 2.69 (1.39)^d^ |

*Note. M* = mean; *SD* = standard deviation. Based on the Bonferroni correction, *p* < .003 indicates significant difference between groups. Means significantly differ from others if they have different superscripts.

Table S30

*The ANOVAs’ Post-hoc Comparisons for the Relationship Profiles in the Eighth Dataset*

| Variables | Age groups | Relationship profiles | | | | | |
| --- | --- | --- | --- | --- | --- | --- | --- |
|  |  | Healthy–independent | Unhealthy–independent | Balanced | Moderate/ ambivalent | Connected | Distant |
|  |  | *M* (*SD*) | *M* (*SD*) | *M* (*SD*) | *M* (*SD*) | *M* (*SD*) | *M* (*SD*) |
| Identity synthesis |  | 3.36 (0.79)^a^ | 2.89 (0.76)^b^ | 3.10 (0.72)^c^ | 2.90 (0.65)^b^ | 3.24 (0.76)^d^ | 2.86 (0.91)^b^ |
| Identity confusion |  | 2.84 (0.81)^a^ | 3.20 (0.70)^b^ | 2.97 (0.70)^c^ | 3.02 (0.62)^c^ | 2.79 (0.73)^a^ | 3.16 (0.81)^b^ |
| Life satisfaction | Early adolescents | 4.31 (1.20)^a^ | 3.03 (1.20)^b^ | 3.85 (1.20)^c^ | 3.54 (1.10)^d^ | 4.33 (1.24)^a^ | 2.86 (1.56)^b^ |
|  | Middle adolescents | 4.05 (1.22)^a^ | 3.14 (1.13)^b^ | 3.67 (1.05)^c^ | 3.52 (1.11)^c^ | 4.03 (1.27)^a^ | 2.84 (1.28)^b^ |
|  | Late adolescents | 4.06 (1.40)^a^ | 3.28 (1.22)^b^ | 3.72 (1.20)^c^ | 3.45 (1.03)^bc^ | 4.07 (1.23)^a^ | 2.82 (1.38)^d^ |
|  | Early emerging adults | 4.01 (1.31)^a^ | 3.05 (1.20)^b^ | 3.62 (1.15)^c^ | 3.39 (1.07)^d^ | 3.83 (1.22)^a^ | 2.84 (1.33)^e^ |
|  | Middle emerging adults | 3.94 (1.38)^a^ | 3.01 (1.25)^b^ | 3.41 (1.22)^c^ | 3.41 (1.05)^c^ | 3.79 (1.30)^a^ | 2.69 (1.40)^d^ |

*Note. M* = mean; *SD* = standard deviation. Based on the Bonferroni correction, *p* < .003 indicates significant difference between groups. Means significantly differ from others if they have different superscripts.

Table S31

*The ANOVAs’ Post-hoc Comparisons for the Relationship Profiles in the Nineth Dataset*

| Variables | Age groups | Relationship profiles | | | | | |
| --- | --- | --- | --- | --- | --- | --- | --- |
|  |  | Healthy–independent | Unhealthy–independent | Balanced | Moderate/ ambivalent | Connected | Distant |
|  |  | *M* (*SD*) | *M* (*SD*) | *M* (*SD*) | *M* (*SD*) | *M* (*SD*) | *M* (*SD*) |
| Identity synthesis |  | 3.37 (0.79)^a^ | 2.89 (0.76)^b^ | 3.10 (0.72)^c^ | 2.90 (0.65)^b^ | 3.24 (0.76)^d^ | 2.86 (0.91)^b^ |
| Identity confusion |  | 2.84 (0.82)^a^ | 3.20 (0.68)^b^ | 2.97 (0.70)^c^ | 3.03 (0.63)^c^ | 2.79 (0.73)^a^ | 3.16 (0.82)^b^ |
| Life satisfaction | Early adolescents | 4.37 (1.21)^a^ | 3.01 (1.19)^b^ | 3.82 (1.15)^c^ | 3.57 (1.12)^c^ | 4.33 (1.24)^a^ | 2.82 (1.53)^b^ |
|  | Middle adolescents | 4.06 (1.21)^a^ | 3.18 (1.07)^b^ | 3.67 (1.09)^c^ | 3.52 (1.15)^c^ | 4.05 (1.29)^a^ | 2.81 (1.31)^d^ |
|  | Late adolescents | 4.08 (1.39)^a^ | 3.22 (1.20)^b^ | 3.74 (1.18)^c^ | 3.48 (1.03)^bc^ | 4.04 (1.25)^a^ | 2.83 (1.39)^d^ |
|  | Early emerging adults | 4.00 (1.30)^a^ | 3.06 (1.18)^b^ | 3.61 (1.16)^c^ | 3.38 (1.08)^d^ | 3.84 (1.21)^a^ | 2.83 (1.34)^e^ |
|  | Middle emerging adults | 3.97 (1.39)^a^ | 3.00 (1.23)^b^ | 3.40 (1.22)^c^ | 3.40 (1.05)^c^ | 3.78 (1.30)^a^ | 2.69 (1.40)^d^ |

*Note. M* = mean; *SD* = standard deviation. Based on the Bonferroni correction, *p* < .003 indicates significant difference between groups. Means significantly differ from others if they have different superscripts.

Table S32

*The ANOVAs’ Post-hoc Comparisons for the Relationship Profiles in the Tenth Dataset*

| Variables | Age groups | Relationship profiles | | | | | |  |
| --- | --- | --- | --- | --- | --- | --- | --- | --- |
|  |  | Healthy–independent | Unhealthy–independent | Balanced | Moderate/ ambivalent | Connected | Distant | |
|  |  | *M* (*SD*) | *M* (*SD*) | *M* (*SD*) | *M* (*SD*) | *M* (*SD*) | *M* (*SD*) | |
| Identity synthesis |  | 3.36 (0.79)^a^ | 2.90 (0.76)^b^ | 3.10 (0.72)^c^ | 2.90 (0.64)^b^ | 3.24 (0.77)^d^ | 2.86 (0.91)^b^ | |
| Identity confusion |  | 2.84 (0.81)^a^ | 3.20 (0.68)^b^ | 2.98 (0.70)^c^ | 3.02 (0.63)^c^ | 2.79 (0.73)^a^ | 3.15 (0.82)^b^ | |
| Life satisfaction | Early adolescents | 4.41 (1.22)^a^ | 3.01 (1.19)^b^ | 3.87 (1.20)^c^ | 3.57 (1.09)^d^ | 4.30 (1.26)^a^ | 2.85 (1.55)^b^ | |
|  | Middle adolescents | 4.06 (1.20)^a^ | 3.23 (1.07)^b^ | 3.64 (1.07)^c^ | 3.49 (1.12)^bc^ | 4.08 (1.26)^a^ | 2.83 (1.31)^d^ | |
|  | Late adolescents | 4.05 (1.38)^a^ | 3.22 (1.24)^b^ | 3.76 (1.18)^cd^ | 3.48 (1.05)^bc^ | 4.05 (1.23)^ad^ | 2.81 (1.38)^e^ | |
|  | Early emerging adults | 4.00 (1.31)^a^ | 3.08 (1.21)^b^ | 3.61 (1.16)^c^ | 3.37 (1.07)^d^ | 3.86 (1.21)^a^ | 2.85 (1.34)^e^ | |
|  | Middle emerging adults | 3.95 (1.40)^a^ | 3.00 (1.23)^b^ | 3.40 (1.22)^c^ | 3.41 (1.05)^c^ | 3.77 (1.30)^a^ | 2.70 (1.39)^d^ | |

*Note. M* = mean; *SD* = standard deviation. Based on the Bonferroni correction, *p* < .003 indicates significant difference between groups. Means significantly differ from others if they have different superscripts.

Table S33

*The ANOVAs’ Post-hoc Comparisons for the Relationship Profiles in the Eleventh Dataset*

| Variables | Age groups | Relationship profiles | | | | | |
| --- | --- | --- | --- | --- | --- | --- | --- |
|  |  | Healthy–independent | Unhealthy–independent | Balanced | Moderate/ ambivalent | Connected | Distant |
|  |  | *M* (*SD*) | *M* (*SD*) | *M* (*SD*) | *M* (*SD*) | *M* (*SD*) | *M* (*SD*) |
| Identity synthesis |  | 3.36 (0.79)^a^ | 2.88 (0.76)^b^ | 3.10 (0.72)^c^ | 2.90 (0.64)^b^ | 3.25 (0.75)^d^ | 2.86 (0.90)^b^ |
| Identity confusion |  | 2.84 (0.82)^a^ | 3.20 (0.69)^b^ | 2.97 (0.70)^c^ | 3.02 (0.63)^c^ | 2.79 (0.73)^a^ | 3.16 (0.82)^b^ |
| Life satisfaction | Early adolescents | 4.40 (1.27)^a^ | 3.04 (1.18)^b^ | 3.80 (1.19)^c^ | 3.55 (1.10)^c^ | 4.33 (1.24)^a^ | 2.87 (1.57)^b^ |
|  | Middle adolescents | 4.13 (1.26)^a^ | 3.21 (1.06)^b^ | 3.63 (1.06)^c^ | 3.49 (1.10)^bc^ | 4.02 (1.27)^a^ | 2.82 (1.29)^d^ |
|  | Late adolescents | 4.06 (1.38)^a^ | 3.19 (1.20)^b^ | 3.75 (1.17)^c^ | 3.45 (1.05)^b^ | 4.07 (1.25)^a^ | 2.85 (1.39)^b^ |
|  | Early emerging adults | 4.00 (1.29)^a^ | 3.04 (1.21)^b^ | 3.62 (1.16)^c^ | 3.39 (1.06)^d^ | 3.83 (1.21)^a^ | 2.86 (1.35)^e^ |
|  | Middle emerging adults | 3.94 (1.39)^a^ | 3.01 (1.24)^b^ | 3.43 (1.22)^c^ | 3.38 (1.06)^c^ | 3.82 (1.26)^a^ | 2.68 (1.40)^d^ |

*Note. M* = mean; *SD* = standard deviation. Based on the Bonferroni correction, *p* < .003 indicates significant difference between groups. Means significantly differ from others if they have different superscripts.

Table S34

*The ANOVAs’ Post-hoc Comparisons for the Relationship Profiles in the Twelfth Dataset*

| Variables | Age groups | Relationship profiles | | | | | |  |
| --- | --- | --- | --- | --- | --- | --- | --- | --- |
|  |  | Healthy–independent | Unhealthy–independent | Balanced | Moderate/ ambivalent | Connected | Distant | |
|  |  | *M* (*SD*) | *M* (*SD*) | *M* (*SD*) | *M* (*SD*) | *M* (*SD*) | *M* (*SD*) | |
| Identity synthesis |  | 3.37 (0.78)^a^ | 2.89 (0.75)^b^ | 3.10 (0.72)^c^ | 2.90 (0.64)^b^ | 3.24 (0.76)^d^ | 2.86 (0.91)^b^ | |
| Identity confusion |  | 2.84 (0.82)^a^ | 3.19 (0.68)^b^ | 2.98 (0.70)^c^ | 3.02 (0.63)^c^ | 2.79 (0.72)^a^ | 3.15 (0.82)^b^ | |
| Life satisfaction | Early adolescents | 4.49 (1.19)^a^ | 3.02 (1.20)^b^ | 3.82 (1.18)^c^ | 3.57 (1.10)^c^ | 4.30 (1.25)^a^ | 2.79 (1.51)^b^ | |
|  | Middle adolescents | 4.07 (1.22)^a^ | 3.17 (1.09)^b^ | 3.64 (1.07)^c^ | 3.47 (1.11)^c^ | 4.06 (1.26)^a^ | 2.85 (1.28)^d^ | |
|  | Late adolescents | 4.06 (1.40)^a^ | 3.22 (1.19)^b^ | 3.77 (1.18)^cd^ | 3.49 (1.05)^bc^ | 4.06 (1.24)^ad^ | 2.84 (1.39)^e^ | |
|  | Early emerging adults | 4.01 (1.31)^a^ | 3.06 (1.22)^b^ | 3.60 (1.15)^c^ | 3.40 (1.06)^d^ | 3.86 (1.21)^a^ | 2.83 (1.34)^e^ | |
|  | Middle emerging adults | 3.93 (1.39)^a^ | 2.99 (1.22)^b^ | 3.42 (1.22)^c^ | 3.40 (1.06)^c^ | 3.81 (1.30)^a^ | 2.70 (1.40)^d^ | |

*Note. M* = mean; *SD* = standard deviation. Based on the Bonferroni correction, *p* < .003 indicates significant difference between groups. Means significantly differ from others if they have different superscripts.

Table S35

*The ANOVAs’ Post-hoc Comparisons for the Relationship Profiles in the Thirteenth Dataset*

| Variables | Age groups | Relationship profiles | | | | | |
| --- | --- | --- | --- | --- | --- | --- | --- |
|  |  | Healthy–independent | Unhealthy–independent | Balanced | Moderate/ ambivalent | Connected | Distant |
|  |  | *M* (*SD*) | *M* (*SD*) | *M* (*SD*) | *M* (*SD*) | *M* (*SD*) | *M* (*SD*) |
| Identity synthesis |  | 3.37 (0.79)^a^ | 2.89 (0.75)^b^ | 3.10 (0.73)^c^ | 2.90 (0.64)^b^ | 3.24 (0.76)^d^ | 2.86 (0.91)^b^ |
| Identity confusion |  | 2.84 (0.82)^a^ | 3.20 (0.69)^b^ | 2.97 (0.70)^c^ | 3.02 (0.63)^c^ | 2.79 (0.72)^a^ | 3.16 (0.82)^b^ |
| Life satisfaction | Early adolescents | 4.42 (1.18)^a^ | 3.04 (1.22)^b^ | 3.81 (1.16)^c^ | 3.58 (1.09)^c^ | 4.31 (1.26)^a^ | 2.85 (1.52)^b^ |
|  | Middle adolescents | 4.03 (1.23)^a^ | 3.20 (1.07)^b^ | 3.67 (1.07)^c^ | 3.47 (1.14)^bc^ | 4.04 (1.27)^a^ | 2.76 (1.34)^d^ |
|  | Late adolescents | 4.07 (1.40)^a^ | 3.23 (1.20)^b^ | 3.74 (1.16)^c^ | 3.44 (1.06)^bc^ | 4.07 (1.25)^a^ | 2.82 (1.39)^d^ |
|  | Early emerging adults | 3.98 (1.30)^a^ | 3.06 (1.19)^b^ | 3.61 (1.16)^c^ | 3.38 (1.08)^d^ | 3.87 (1.23)^a^ | 2.83 (1.33)^e^ |
|  | Middle emerging adults | 3.96 (1.38)^a^ | 2.99 (1.24)^b^ | 3.40 (1.22)^c^ | 3.42 (1.04)^c^ | 3.81 (1.28)^a^ | 2.69 (1.39)^d^ |

*Note. M* = mean; *SD* = standard deviation. Based on the Bonferroni correction, *p* < .003 indicates significant difference between groups. Means significantly differ from others if they have different superscripts.

Table S36

*The ANOVAs’ Post-hoc Comparisons for the Relationship Profiles in the Fourteenth Dataset*

| Variables | Age groups | Relationship profiles | | | | | |
| --- | --- | --- | --- | --- | --- | --- | --- |
|  |  | Healthy–independent | Unhealthy–independent | Balanced | Moderate/ ambivalent | Connected | Distant |
|  |  | *M* (*SD*) | *M* (*SD*) | *M* (*SD*) | *M* (*SD*) | *M* (*SD*) | *M* (*SD*) |
| Identity synthesis |  | 3.35 (0.79)^a^ | 2.89 (0.76)^b^ | 3.10 (0.72)^c^ | 2.90 (0.64)^b^ | 3.25 (0.75)^d^ | 2.85 (0.91)^b^ |
| Identity confusion |  | 2.84 (0.81)^a^ | 3.20 (0.69)^b^ | 2.98 (0.70)^c^ | 3.02 (0.63)^c^ | 2.79 (0.73)^a^ | 3.16 (0.82)^b^ |
| Life satisfaction | Early adolescents | 4.30 (1.25)^a^ | 3.03 (1.20)^b^ | 3.82 (1.21)^c^ | 3.57 (1.11)^c^ | 4.32 (1.25)^a^ | 2.86 (1.58)^b^ |
|  | Middle adolescents | 4.06 (1.27)^a^ | 3.16 (1.08)^b^ | 3.64 (1.07)^c^ | 3.50 (1.13)^c^ | 4.04 (1.26)^a^ | 2.83 (1.31)^d^ |
|  | Late adolescents | 4.05 (1.39)^a^ | 3.20 (1.20)^b^ | 3.76 (1.19)^c^ | 3.48 (1.03)^bc^ | 4.07 (1.25)^a^ | 2.83 (1.39)^d^ |
|  | Early emerging adults | 3.99 (1.31)^a^ | 3.06 (1.20)^b^ | 3.61 (1.15)^c^ | 3.37 (1.07)^d^ | 3.86 (1.22)^a^ | 2.84 (1.34)^e^ |
|  | Middle emerging adults | 3.95 (1.39)^a^ | 3.01 (1.24)^b^ | 3.41 (1.22)^c^ | 3.42 (1.03)^c^ | 3.81 (1.28)^a^ | 2.67 (1.39)^d^ |

*Note. M* = mean; *SD* = standard deviation. Based on the Bonferroni correction, *p* < .003 indicates significant difference between groups. Means significantly differ from others if they have different superscripts.

Table S37

*The ANOVAs’ Post-hoc Comparisons for the Relationship Profiles in the Fifteenth Dataset*

| Variables | Age groups | Relationship profiles | | | | | |
| --- | --- | --- | --- | --- | --- | --- | --- |
|  |  | Healthy–independent | Unhealthy–independent | Balanced | Moderate/ ambivalent | Connected | Distant |
|  |  | *M* (*SD*) | *M* (*SD*) | *M* (*SD*) | *M* (*SD*) | *M* (*SD*) | *M* (*SD*) |
| Identity synthesis |  | 3.36 (0.79)^a^ | 2.89 (0.76)^b^ | 3.10 (0.72)^c^ | 2.90 (0.63)^b^ | 3.25 (0.76)^d^ | 2.86 (0.91)^b^ |
| Identity confusion |  | 2.84 (0.82)^a^ | 3.20 (0.69)^b^ | 2.98 (0.70)^c^ | 3.02 (0.62)^c^ | 2.79 (0.73)^a^ | 3.16 (0.82)^b^ |
| Life satisfaction | Early adolescents | 4.37 (1.23)^a^ | 3.07 (1.23)^b^ | 3.83 (1.16)^c^ | 3.55 (1.11)^c^ | 4.32 (1.26)^a^ | 2.84 (1.57)^b^ |
|  | Middle adolescents | 4.03 (1.23)^a^ | 3.19 (1.06)^b^ | 3.67 (1.09)^c^ | 3.53 (1.10)^c^ | 4.05 (1.27)^a^ | 2.85 (1.35)^d^ |
|  | Late adolescents | 4.06 (1.40)^a^ | 3.24 (1.23)^b^ | 3.74 (1.18)^c^ | 3.49 (1.01)^bc^ | 4.05 (1.25)^a^ | 2.82 (1.37)^d^ |
|  | Early emerging adults | 3.99 (1.31)^a^ | 3.05 (1.20)^b^ | 3.60 (1.17)^c^ | 3.39 (1.06)^d^ | 3.84 (1.21)^a^ | 2.84 (1.35)^e^ |
|  | Middle emerging adults | 3.95 (1.38)^a^ | 3.01 (1.23)^b^ | 3.42 (1.22)^c^ | 3.40 (1.04)^c^ | 3.79 (1.30)^a^ | 2.69 (1.40)^d^ |

*Note. M* = mean; *SD* = standard deviation. Based on the Bonferroni correction, *p* < .003 indicates significant difference between groups. Means significantly differ from others if they have different superscripts.

Table S38

*The ANOVAs’ Post-hoc Comparisons for the Relationship Profiles in the Sixteenth Dataset*

| Variables | Age groups | Relationship profiles | | | | | |
| --- | --- | --- | --- | --- | --- | --- | --- |
|  |  | Healthy–independent | Unhealthy–independent | Balanced | Moderate/ ambivalent | Connected | Distant |
|  |  | *M* (*SD*) | *M* (*SD*) | *M* (*SD*) | *M* (*SD*) | *M* (*SD*) | *M* (*SD*) |
| Identity synthesis |  | 3.35 (0.79)^a^ | 2.88 (0.76)^b^ | 3.10 (0.72)^c^ | 2.90 (0.64)^b^ | 3.25 (0.76)^d^ | 2.86 (0.91)^b^ |
| Identity confusion |  | 2.85 (0.82)^a^ | 3.20 (0.69)^b^ | 2.97 (0.70)^c^ | 3.02 (0.63)^c^ | 2.78 (0.73)^d^ | 3.16 (0.82)^b^ |
| Life satisfaction | Early adolescents | 4.31 (1.28)^a^ | 3.06 (1.20)^b^ | 3.80 (1.20)^c^ | 3.56 (1.09)^c^ | 4.34 (1.24)^a^ | 2.88 (1.59)^b^ |
|  | Middle adolescents | 4.03 (1.18)^a^ | 3.22 (1.10)^b^ | 3.64 (1.09)^c^ | 3.49 (1.12)^bc^ | 4.09 (1.28)^a^ | 2.83 (1.29)^d^ |
|  | Late adolescents | 4.08 (1.39)^a^ | 3.24 (1.20)^b^ | 3.74 (1.17)^c^ | 3.46 (1.07)^bc^ | 4.06 (1.24)^a^ | 2.82 (1.36)^d^ |
|  | Early emerging adults | 3.99 (1.31)^a^ | 3.04 (1.20)^b^ | 3.61 (1.15)^c^ | 3.40 (1.08)^d^ | 3.85 (1.21)^a^ | 2.83 (1.34)^e^ |
|  | Middle emerging adults | 3.96 (1.38)^a^ | 3.01 (1.24)^b^ | 3.41 (1.21)^c^ | 3.39 (1.04)^c^ | 3.79 (1.30)^a^ | 2.68 (1.40)^d^ |

*Note. M* = mean; *SD* = standard deviation. Based on the Bonferroni correction, *p* < .003 indicates significant difference between groups. Means significantly differ from others if they have different superscripts.

Table S39

*The ANOVAs’ Post-hoc Comparisons for the Relationship Profiles in the Seventeenth Dataset*

| Variables | Age groups | Relationship profiles | | | | | |
| --- | --- | --- | --- | --- | --- | --- | --- |
|  |  | Healthy–independent | Unhealthy–independent | Balanced | Moderate/ ambivalent | Connected | Distant |
|  |  | *M* (*SD*) | *M* (*SD*) | *M* (*SD*) | *M* (*SD*) | *M* (*SD*) | *M* (*SD*) |
| Identity synthesis |  | 3.36 (0.79)^a^ | 2.89 (0.75)^b^ | 3.10 (0.72)^c^ | 2.90 (0.64)^b^ | 3.25 (0.76)^d^ | 2.85 (0.91)^b^ |
| Identity confusion |  | 2.84 (0.82)^a^ | 3.20 (0.68)^b^ | 2.97 (0.70)^c^ | 3.02 (0.63)^c^ | 2.79 (0.73)^a^ | 3.16 (0.82)^b^ |
| Life satisfaction | Early adolescents | 4.41 (1.23)^a^ | 3.04 (1.22)^b^ | 3.85 (1.20)^c^ | 3.57 (1.10)^c^ | 4.31 (1.25)^a^ | 2.82 (1.50)^b^ |
|  | Middle adolescents | 4.05 (1.24)^a^ | 3.23 (1.05)^b^ | 3.65 (1.08)^c^ | 3.50 (1.13)^bc^ | 4.04 (1.30)^a^ | 2.79 (1.29)^d^ |
|  | Late adolescents | 4.03 (1.42)^a^ | 3.25 (1.20)^b^ | 3.74 (1.19)^c^ | 3.48 (1.05)^bc^ | 4.05 (1.25)^a^ | 2.80 (1.38)^d^ |
|  | Early emerging adults | 3.99 (1.30)^a^ | 3.05 (1.21)^b^ | 3.62 (1.15)^c^ | 3.37 (1.08)^d^ | 3.84 (1.23)^a^ | 2.85 (1.34)^e^ |
|  | Middle emerging adults | 3.97 (1.38)^a^ | 2.99 (1.24)^b^ | 3.41 (1.22)^c^ | 3.40 (1.05)^c^ | 3.81 (1.28)^a^ | 2.70 (1.39)^d^ |

*Note. M* = mean; *SD* = standard deviation. Based on the Bonferroni correction, *p* < .003 indicates significant difference between groups. Means significantly differ from others if they have different superscripts.

Table S40

*The ANOVAs’ Post-hoc Comparisons for the Relationship Profiles in the Eighteenth Dataset*

| Variables | Age groups | Relationship profiles | | | | | |
| --- | --- | --- | --- | --- | --- | --- | --- |
|  |  | Healthy–independent | Unhealthy–independent | Balanced | Moderate/ ambivalent | Connected | Distant |
|  |  | *M* (*SD*) | *M* (*SD*) | *M* (*SD*) | *M* (*SD*) | *M* (*SD*) | *M* (*SD*) |
| Identity synthesis |  | 3.36 (0.79)^a^ | 2.88 (0.75)^b^ | 3.10 (0.72)^c^ | 2.90 (0.64)^b^ | 3.25 (0.76)^d^ | 2.86 (0.90)^b^ |
| Identity confusion |  | 2.84 (0.81)^a^ | 3.20 (0.68)^b^ | 2.97 (0.70)^c^ | 3.02 (0.63)^c^ | 2.79 (0.73)^a^ | 3.15 (0.81)^b^ |
| Life satisfaction | Early adolescents | 4.33 (1.21)^a^ | 2.99 (1.20)^b^ | 3.81 (1.20)^c^ | 3.57 (1.09)^c^ | 4.33 (1.24)^a^ | 2.86 (1.59)^b^ |
|  | Middle adolescents | 4.06 (1.23)^a^ | 3.18 (1.05)^b^ | 3.62 (1.07)^c^ | 3.52 (1.16)^c^ | 4.06 (1.26)^a^ | 2.88 (1.33)^b^ |
|  | Late adolescents | 4.06 (1.38)^a^ | 3.21 (1.19)^b^ | 3.77 (1.20)^c^ | 3.51 (1.05)^bc^ | 4.08 (1.24)^a^ | 2.84 (1.38)^d^ |
|  | Early emerging adults | 3.99 (1.30)^a^ | 3.05 (1.20)^b^ | 3.60 (1.17)^c^ | 3.40 (1.07)^d^ | 3.85 (1.23)^a^ | 2.83 (1.34)^e^ |
|  | Middle emerging adults | 3.94 (1.40)^a^ | 2.99 (1.24)^b^ | 3.40 (1.21)^c^ | 3.41 (1.06)^c^ | 3.81 (1.29)^a^ | 2.70 (1.39)^d^ |

*Note. M* = mean; *SD* = standard deviation. Based on the Bonferroni correction, *p* < .003 indicates significant difference between groups. Means significantly differ from others if they have different superscripts.

Table S41

*The ANOVAs’ Post-hoc Comparisons for the Relationship Profiles in the Nineteenth Dataset*

| Variables | Age groups | Relationship profiles | | | | | |  |
| --- | --- | --- | --- | --- | --- | --- | --- | --- |
|  |  | Healthy–independent | Unhealthy–independent | Balanced | Moderate/ ambivalent | Connected | Distant | |
|  |  | *M* (*SD*) | *M* (*SD*) | *M* (*SD*) | *M* (*SD*) | *M* (*SD*) | *M* (*SD*) | |
| Identity synthesis |  | 3.36 (0.79)^a^ | 2.89 (0.76)^b^ | 3.10 (0.72)^c^ | 2.91 (0.63)^b^ | 3.24 (0.77)^d^ | 2.86 (0.91)^b^ | |
| Identity confusion |  | 2.84 (0.82)^a^ | 3.20 (0.69)^b^ | 2.97 (0.70)^c^ | 3.01 (0.62)^c^ | 2.79 (0.73)^a^ | 3.15 (0.82)^b^ | |
| Life satisfaction | Early adolescents | 4.34 (1.28)^a^ | 3.01 (1.19)^b^ | 3.82 (1.19)^c^ | 3.58 (1.11)^c^ | 4.31 (1.26)^a^ | 2.87 (1.58)^b^ | |
|  | Middle adolescents | 4.07 (1.20)^a^ | 3.15 (1.09)^b^ | 3.65 (1.09)^c^ | 3.49 (1.13)^c^ | 4.06 (1.29)^a^ | 2.86 (1.32)^b^ | |
|  | Late adolescents | 4.06 (1.40)^a^ | 3.24 (1.20)^b^ | 3.75 (1.19)^cd^ | 3.45 (1.04)^bc^ | 4.05 (1.24)^ad^ | 2.84 (1.39)^e^ | |
|  | Early emerging adults | 4.00 (1.29)^a^ | 3.04 (1.21)^b^ | 3.62 (1.16)^c^ | 3.39 (1.06)^d^ | 3.84 (1.23)^a^ | 2.84 (1.33)^e^ | |
|  | Middle emerging adults | 3.95 (1.39)^a^ | 3.01 (1.24)^b^ | 3.40 (1.22)^c^ | 3.42 (1.04)^c^ | 3.80 (1.30)^a^ | 2.69 (1.40)^d^ | |

*Note. M* = mean; *SD* = standard deviation. Based on the Bonferroni correction, *p* < .003 indicates significant difference between groups. Means significantly differ from others if they have different superscripts.

Table S42

*The ANOVAs’ Post-hoc Comparisons for the Relationship Profiles in the Twentieth Dataset*

| Variables | Age groups | Relationship profiles | | | | | |
| --- | --- | --- | --- | --- | --- | --- | --- |
|  |  | Healthy–independent | Unhealthy–independent | Balanced | Moderate/ ambivalent | Connected | Distant |
|  |  | *M* (*SD*) | *M* (*SD*) | *M* (*SD*) | *M* (*SD*) | *M* (*SD*) | *M* (*SD*) |
| Identity synthesis |  | 3.36 (0.79)^a^ | 2.89 (0.75)^b^ | 3.10 (0.72)^c^ | 2.90 (0.64)^b^ | 3.25 (0.76)^d^ | 2.86 (0.91)^b^ |
| Identity confusion |  | 2.84 (0.81)^a^ | 3.20 (0.69)^b^ | 2.97 (0.70)^c^ | 3.02 (0.63)^c^ | 2.79 (0.73)^a^ | 3.16 (0.82)^b^ |
| Life satisfaction | Early adolescents | 4.48 (1.27)^a^ | 3.03 (1.21)^b^ | 3.83 (1.17)^c^ | 3.58 (1.11)^c^ | 4.31 (1.25)^a^ | 2.90 (1.57)^b^ |
|  | Middle adolescents | 4.00 (1.23)^a^ | 3.18 (1.07)^b^ | 3.66 (1.07)^c^ | 3.48 (1.14)^c^ | 4.06 (1.26)^a^ | 2.87 (1.29)^b^ |
|  | Late adolescents | 4.05 (1.39)^a^ | 3.22 (1.19)^b^ | 3.74 (1.18)^c^ | 3.46 (1.05)^bc^ | 4.09 (1.24)^a^ | 2.84 (1.39)^d^ |
|  | Early emerging adults | 4.01 (1.30)^a^ | 3.07 (1.20)^b^ | 3.61 (1.16)^c^ | 3.38 (1.07)^d^ | 3.87 (1.23)^a^ | 2.85 (1.34)^e^ |
|  | Middle emerging adults | 3.96 (1.38)^a^ | 3.00 (1.24)^b^ | 3.40 (1.22)^c^ | 3.40 (1.04)^c^ | 3.78 (1.31)^a^ | 2.71 (1.40)^d^ |

*Note. M* = mean; *SD* = standard deviation. Based on the Bonferroni correction, *p* < .003 indicates significant difference between groups. Means significantly differ from others if they have different superscripts.

**Online Resource 4**

**Sensitivity Analyses**

A series of sensitivity analyses were performed to examine the robustness of the results of the main analyses. The main analyses (age differences in the distributions of relationship profiles and associations between relationship profiles and psychosocial adjustment) were conducted with gender and residential area as moderators.

**Age Differences in the Distributions of Relationship Profiles**

To examine the influence of gender on age differences in the distributions of relationship profiles, two chi-square tests were conducted for boys/men and girls/women separately. Results were statistically significant for both boys/men (χ^2^ (20, *N* = 6,467) = 472.14–522.31, *p*s < .001, Cramer’s *V*s = .14, *p*s < .001; pooled *F* (20, 1,705.15) = 22.54, *p* < .001) and girls/women (χ^2^ (20, *N* = 7,951) = 415.89–453.26, *p*s < .001, Cramer’s *V*s = .11–.12, *p*s < .001; pooled *F* (20, 4,131.99) = 20.64, *p* < .001). As shown in Tables S43 and S44, residual analyses indicated that the *connected* profile was the most prevalent in younger age groups. Furthermore, the *healthy–independent* and *distant* profiles were overrepresented in older age groups. Thus, the original results were generally replicated for both boys/men and girls/women.

To understand the influence of residential areas on age differences in the distributions of relationship profiles, two additional chi-square tests were performed for those living in urban and rural areas. These showed significant results in both those living in relatively urban (χ^2^ (10, *N* = 11,503) = 826.89–869.32, *p*s < .001, Cramer’s *V*s = .13–.14, *p*s < .001; pooled *F* (10, 7,397.56) = 81.17, *p* < .001) and rural areas (χ^2^ (10, *N* = 2,922) = 24.27–37.49, *p*s = .000–.007, Cramer’s *V*s = .09–.11, *p*s = .000–.007; pooled *F* (10, 1,750.12) = 2.59, *p* = .004). Regarding those living in relatively urban areas (see Table S45), the *connected* profile was prevalent among early adolescents. Furthermore, late adolescents were overrepresented in the *healthy–independent* profile; in early and middle emerging adults, the *healthy–independent* and *distant* profiles were predominant. These results confirm the original results. Regarding those living in relatively rural areas (see Table S46), although such individuals were not included in early and middle adolescent samples, the original results were generally replicated. Specifically, relatively younger participants (late adolescents) were underrepresented in the *healthy–independent* profile, while relatively older participants (early emerging adults) were overrepresented.

**Age Differences in the Associations Between Relationship Profiles and Psychosocial Adjustment**

The influence of gender and residential area on age differences in the associations between the relationship profiles and psychosocial adjustment were examined by a series of ANOVAs for each outcome. The independent variables were relationship profile, age, gender, residential area, and interactions consisting of all patterns of their combination; the dependent variables were identity synthesis, identity confusion, or life satisfaction (see Table S47). Regarding identity synthesis, the original finding (main effect of the relationship profile) was moderated by gender (*F* (5, 6,993.39) = 2.39, *p* = .036, partial η^2^ = .001) but not by residential area (*F* (5, 2,786.57) = 0.94, *p* = .451, partial η^2^ = .000). For both boys/men and girls/women, post-hoc Bonferroni-adjusted tests indicated that the *healthy–independent* profile scored the highest on identity synthesis, followed by the *connected*, *balanced*, *moderate/ambivalent*, *unhealthy–independent*, and *distant* profiles. These results were consistent with the original results.

Regarding identity confusion, the original finding (main effect of the relationship profile) was moderated by gender (*F* (5, 1,262.04) = 3.92, *p* = .002, partial η^2^ = .002) but not by residential area (*F* (5, 2,624.21) = 1.31, *p* = .257, partial η^2^ = .000). Table S48 reports results of post-hoc Bonferroni-adjusted tests. Among boys/men, the scores of identity confusion were highest in the *unhealthy–independent*, followed by the *distant*, *moderate/ambivalent*, *balanced*, *healthy*–*independent*, and *connected* profiles. Although a couple of significant differences were not consistent with the original (e.g., boys/men in the *unhealthy–independent* profile scored higher on identity confusion than boys/men in the *distant* profile did), their effect sizes were small (Cohen’s *d*s = 0.18–0.19). For girls/women, consistent with the original results, the identity confusion scores were highest in the *unhealthy–independent* and *distant* profiles, followed by the *moderate/ambivalent*, *balanced*, *healthy*–*independent*, and *connected* profiles.

As for life satisfaction, the original finding (interaction effect of relationship profile and age group) was not moderated by gender (*F* (20, 684.99) = 0.86, *p* = .633, partial η^2^ = .002) or residential area (*F* (10, 3,607.40) = 0.55, *p* = .856, partial η^2^ = .000).

Taken together, the main study results were generally confirmed when participants’ gender and residential area were considered to be moderators. This highlights the robustness of the results of the main analyses.

Table S43

*Cross-tabulation of Relationship Profiles and Age Groups Among Boys/Men*

| Age groups | Indices | Relationship profiles | | | | | | Total (%) |
| --- | --- | --- | --- | --- | --- | --- | --- | --- |
|  |  | Healthy–independent | Unhealthy–independent | Balanced | Moderate/ ambivalent | Connected | Distant |  |
| Early adolescents | Frequency | **3.6 (−)** | **13.9 (−)** | **15.5 (−)** | **30.4 (+)** | **28.3 (+)** | **8.3 (−)** | 100 |
|  | ASR | **−8.4** | **−2.7** | **−8.9** | **8.0** | **16.3** | **−3.3** |  |
|  |  |  |  |  |  |  |  |  |
| Middle adolescents | Frequency | 11.0 | **19.2 (+)** | **29.6 (+)** | **16.7 (−)** | **14.7 (+)** | **8.8 (−)** | 100 |
|  | ASR | −0.3 | **2.5** | **2.2** | **−4.0** | **2.3** | **−3.1** |  |
|  |  |  |  |  |  |  |  |  |
| Late adolescents | Frequency | **18.3 (+)** | 16.3 | 27.1 | **17.9 (−)** | **10.2 (−)** | 10.2 | 100 |
|  | ASR | **5.9** | −0.3 | 0.1 | **−2.0** | **−2.0** | −1.0 |  |
|  |  |  |  |  |  |  |  |  |
| Early emerging adults | Frequency | 12.0 | 16.6 | **29.6 (+)** | 19.8 | **8.5 (−)** | **13.6 (+)** | 100 |
|  | ASR | 1.2 | −0.3 | **2.9** | −1.5 | **−6.2** | **3.6** |  |
|  |  |  |  |  |  |  |  |  |
| Middle emerging adults | Frequency | **12.5 (+)** | 17.2 | **29.0 (+)** | 20.9 | **7.6 (−)** | **12.8 (+)** | 100 |
|  | ASR | **2.0** | 0.6 | **2.3** | −0.2 | **−7.6** | **2.4** |  |
|  |  |  |  |  |  |  |  |  |
| Total (%) |  | 11.2 | 16.8 | 27.0 | 21.0 | 12.7 | 11.3 | 100 |

*Note.* Observed values indicated in bold are significantly different from expected values (tested by using adjusted residual analysis); (+) indicates that the observed value is higher than the expected value; (−) indicates that the observed value is lower than the expected value.

ASR = adjusted standardized residual.

Table S44

*Cross-tabulation of Relationship Profiles and Age Groups Among Girls/Women*

| Age groups | Indices | Relationship profiles | | | | | | Total (%) |
| --- | --- | --- | --- | --- | --- | --- | --- | --- |
|  |  | Healthy–independent | Unhealthy–independent | Balanced | Moderate/ ambivalent | Connected | Distant |  |
| Early adolescents | Frequency | **7.0 (−)** | **11.7 (−)** | **14.4 (−)** | **25.1 (+)** | **34.8 (+)** | **6.9 (−)** | 100 |
|  | ASR | **−7.4** | **−3.8** | **−6.2** | **9.4** | **15.5** | **−5.6** |  |
|  |  |  |  |  |  |  |  |  |
| Middle adolescents | Frequency | **20.4 (+)** | 17.3 | **27.2 (+)** | **10.1 (−)** | 13.5 | 11.5 | 100 |
|  | ASR | **2.3** | 0.3 | **2.1** | **−2.6** | −1.1 | −1.7 |  |
|  |  |  |  |  |  |  |  |  |
| Late adolescents | Frequency | 17.9 | 18.1 | 21.8 | 13.1 | **17.5 (+)** | **11.5 (−)** | 100 |
|  | ASR | 1.0 | 1.1 | −1.5 | −0.5 | **2.3** | **−2.2** |  |
|  |  |  |  |  |  |  |  |  |
| Early emerging adults | Frequency | **18.0 (+)** | 16.6 | 24.6 | 12.9 | **12.2 (−)** | **15.6 (+)** | 100 |
|  | ASR | **2.0** | −0.4 | 1.4 | −1.4 | **−5.3** | **3.6** |  |
|  |  |  |  |  |  |  |  |  |
| Middle emerging adults | Frequency | 17.2 | 17.8 | **25.2 (+)** | **12.4 (−)** | **12.5 (−)** | **14.9 (+)** | 100 |
|  | ASR | 0.6 | 1.8 | **2.3** | **−2.4** | **−4.9** | **2.2** |  |
|  |  |  |  |  |  |  |  |  |
| Total (%) |  | 16.9 | 16.8 | 23.7 | 13.7 | 15.1 | 13.8 | 100 |

*Note.* Observed values indicated in bold are significantly different from expected values (tested by using adjusted residual analysis); (+) indicates that the observed value is higher than the expected value; (−) indicates that the observed value is lower than the expected value.

ASR = adjusted standardized residual.

Table S45

*Cross-tabulation of Relationship Profiles and Age Groups Among Those Living in Relatively Urban Areas*

| Age groups | Indices | Relationship profiles | | | | | | Total (%) |
| --- | --- | --- | --- | --- | --- | --- | --- | --- |
|  |  | Healthy–independent | Unhealthy–independent | Balanced | Moderate/ ambivalent | Connected | Distant |  |
| Early adolescents | Frequency | **5.3 (−)** | **13.0 (−)** | **14.7 (−)** | **28.1 (+)** | **31.1 (+)** | **7.8 (−)** | 100 |
|  | ASR | **−11.5** | **−4.5** | **−10.6** | **12.9** | **20.6** | **−6.0** |  |
|  |  |  |  |  |  |  |  |  |
| Middle adolescents | Frequency | 13.6 | **18.6 (+)** | **28.5 (+)** | **14.6 (−)** | 14.9 | **9.7 (−)** | 100 |
|  | ASR | −0.7 | **2.4** | **4.0** | **−3.3** | 0.0 | **−3.5** |  |
|  |  |  |  |  |  |  |  |  |
| Late adolescents | Frequency | **19.1 (+)** | 16.8 | 24.3 | **13.6 (−)** | 15.2 | 10.9 | 100 |
|  | ASR | **5.8** | 0.1 | −0.5 | **−4.0** | 0.4 | −1.5 |  |
|  |  |  |  |  |  |  |  |  |
| Early emerging adults | Frequency | **15.2 (+)** | 17.3 | **26.5 (+)** | **16.0 (−)** | **10.8 (−)** | **14.2 (+)** | 100 |
|  | ASR | **2.0** | 1.1 | **2.6** | **−2.3** | **−7.7** | **4.2** |  |
|  |  |  |  |  |  |  |  |  |
| Middle emerging adults | Frequency | **15.9 (+)** | 16.9 | **26.7 (+)** | **16.0 (−)** | **10.1 (−)** | **14.4 (+)** | 100 |
|  | ASR | **3.4** | 0.4 | **3.0** | **−2.4** | **−9.1** | **4.6** |  |
|  |  |  |  |  |  |  |  |  |
| Total (%) |  | 14.1 | 16.7 | 24.8 | 17.3 | 14.9 | 12.2 | 100 |

*Note.* Observed values indicated in bold are significantly different from expected values (tested by using adjusted residual analysis); (+) indicates that the observed value is higher than the expected value; (−) indicates that the observed value is lower than the expected value.

ASR = adjusted standardized residual.

Table S46

*Cross-tabulation of Relationship Profiles and Age Groups Among Those Living in Relatively Rural Areas*

| Age groups | Indices | Relationship profiles | | | | | | Total (%) |
| --- | --- | --- | --- | --- | --- | --- | --- | --- |
|  |  | Healthy–independent | Unhealthy–independent | Balanced | Moderate/ ambivalent | Connected | Distant |  |
| Late adolescents | Frequency | **8.1 (−)** | 19.3 | **17.8 (−)** | **27.4 (+)** | 13.3 | 14.1 | 100 |
|  | ASR | **−2.4** | 0.8 | **−2.1** | **3.7** | 0.7 | −0.3 |  |
|  |  |  |  |  |  |  |  |  |
| Early emerging adults | Frequency | **16.9 (+)** | 15.4 | 25.9 | 15.0 | 10.9 | 15.9 | 100 |
|  | ASR | **2.2** | −1.8 | 0.4 | −1.4 | −0.8 | 1.3 |  |
|  |  |  |  |  |  |  |  |  |
| Middle emerging adults | Frequency | 14.5 | 17.7 | 25.9 | 15.9 | 11.8 | 14.2 | 100 |
|  | ASR | −1.2 | 1.5 | 0.4 | −0.2 | 0.5 | −1.2 |  |
|  |  |  |  |  |  |  |  |  |
| Total (%) |  | 15.4 | 16.7 | 25.5 | 16.0 | 11.4 | 15.0 | 100 |

*Note.* Observed values indicated in bold are significantly different from expected values (tested by using adjusted residual analysis); (+) indicates that the observed value is higher than the expected value; (−) indicates that the observed value is lower than the expected value.

ASR = adjusted standardized residual.

Table S47

*The ANOVA Results for Identity Synthesis*

| Dependent variable | Independent variables | *F*-values | Partial η^2^ |
| --- | --- | --- | --- |
| Identity synthesis | Relational profile | *F* (5, 7,545.13) = 157.08*** | .054 |
|  | Age group | *F* (4, 12,671.984) = 10.52*** | .003 |
|  | Gender | *F* (1, 6,578.95) = 178.91*** | .012 |
|  | Residential area | *F* (1, 10,986.94) = 8.21** | .001 |
|  | Relational profile × Age group | *F* (20, 2,025.94) = 1.39 | .002 |
|  | Relational profile × Gender | *F* (5, 6,993.39) = 2.39* | .001 |
|  | Age group × Gender | *F* (4, 6,317.65) = 1.17 | .000 |
|  | Relational profile × Residential area | *F* (5, 2,786.57) = 0.94 | .000 |
|  | Age group × Residential area | *F* (2, 14,814.61) = 2.10 | .000 |
|  | Gender × Residential area | *F* (1, 11,435.64) = 1.05 | .000 |
|  | Relational profile × Age group × Gender | *F* (20, 3,308.43) = 0.68 | .001 |
|  | Relational profile × Age group × Residential area | *F* (10, 3,761.89) = 0.76 | .001 |
|  | Relational profile × Gender × Residential area | *F* (5, 3,930.98) = 0.42 | .000 |
|  | Age group × Gender × Residential area | *F* (2, 18,213.15) = 1.19 | .000 |
|  | Relational profile × Age group × Gender × Residential area | *F* (10, 1,387.51) = 0.86 | .001 |
| Identity confusion | Relational profile | *F* (5, 14,994.29) = 108.96*** | .038 |
|  | Age group | *F* (4, 15,361.05) = 120.55*** | .034 |
|  | Gender | *F* (1, 22,830.85) = 4.95* | .000 |
|  | Residential area | *F* (1, 35,960.80) = 11.38*** | .001 |
|  | Relational profile × Age group | *F* (20, 902.84) = 1.20 | .002 |
|  | Relational profile × Gender | *F* (5, 1,262.04) = 3.92** | .002 |
|  | Age group × Gender | *F* (4, 133,785.96) = 0.33 | .000 |
|  | Relational profile × Residential area | *F* (5, 2,624.21) = 1.31 | .001 |
|  | Age group × Residential area | *F* (2, 3,481.06) = 0.21 | .000 |
|  | Gender × Residential area | *F* (1, 588,666.92) = 2.14 | .000 |
|  | Relational profile × Age group × Gender | *F* (20, 4,892.19) = 0.43 | .001 |
|  | Relational profile × Age group × Residential area | *F* (10, 4,602.05) = 0.69 | .001 |
|  | Relational profile × Gender × Residential area | *F* (5, 10,203.13) = 0.37 | .000 |
|  | Age group × Gender × Residential area | *F* (2, 36,624.42) = 0.73 | .000 |
|  | Relational profile × Age group × Gender × Residential area | *F* (10, 2,281.97) = 1.24 | .001 |
| Life satisfaction | Relational profile | *F* (5, 4,139.35) = 312.26*** | .104 |
|  | Age group | *F* (4, 26,692.87) = 23.28*** | .007 |
|  | Gender | *F* (1,20,882.48) = 7.64** | .001 |
|  | Residential area | *F* (1, 16,300.85) = 18.02*** | .001 |
|  | Relational profile × Age group | *F* (20, 8,423.03) = 2.13** | .003 |
|  | Relational profile × Gender | *F* (5, 1,488.97) = 2.71* | .001 |
|  | Age group × Gender | *F* (4, 38,656.67) = 1.82 | .001 |
|  | Relational profile × Residential area | *F* (5, 2,677.11) = 0.55 | .000 |
|  | Age group × Residential area | *F* (2, 11,597.77) = 1.33 | .000 |
|  | Gender × Residential area | *F* (1, 14,333.43) = 0.15 | .000 |
|  | Relational profile × Age group × Gender | *F* (20, 684.99) = 0.86 | .002 |
|  | Relational profile × Age group × Residential area | *F* (10, 3,607.40) = 0.55 | .000 |
|  | Relational profile × Gender × Residential area | *F* (5, 3,645.41) = 1.12 | .000 |
|  | Age group × Gender × Residential area | *F* (2, 12,427.41) = 2.61 | .000 |
|  | Relational profile × Age group × Gender × Residential area | *F* (10, 935.03) = 0.78 | .001 |

*Note. M* = mean; *SD* = standard deviation.

**p* < .050, ***p* < .010, ****p* < .001.

Table S48

*The ANOVAs’ Post-hoc Comparisons for Identity Synthesis and Confusion*

| Variables | Groups | Relationship profiles | | | | | | | *F*-values | Partial η^2^ |
| --- | --- | --- | --- | --- | --- | --- | --- | --- | --- | --- |
|  |  | Healthy–independent | Unhealthy–independent | Balanced | Moderate/ ambivalent | Connected | Distant |  | |  |
|  |  | *M* (*SD*) | *M* (*SD*) | *M* (*SD*) | *M* (*SD*) | *M* (*SD*) | *M* (*SD*) |  | |  |
| Identity synthesis | Boys/Men | 3.53 (0.81)^a^ | 2.97 (0.76)^b^ | 3.18 (0.72)^c^ | 2.96 (0.63)^b^ | 3.35 (0.75)^d^ | 3.01 (0.94)^b^ | *F* (5, 4,468.50) = 77.72*** | | .060 |
|  | Girls/Women | 3.27 (0.77)^a^ | 2.81 (0.75)^b^ | 3.01 (0.71)^c^ | 2.83 (0.64)^b^ | 3.17 (0.75)^d^ | 2.76 (0.88)^b^ | *F* (5, 8,446.44  = 91.48*** | | .057 |
| Identity confusion | Boys/Men | 2.92 (0.84)^a^ | 3.19 (0.69)^b^ | 2.97 (0.70)^a^ | 2.99 (0.62)^ad^ | 2.78 (0.74)^c^ | 3.07 (0.83)^d^ | *F* (5, 2,163.82) = 31.07*** | | .026 |
|  | Girls/Women | 2.81 (0.79)^a^ | 3.21 (0.69)^b^ | 2.98 (0.70)^c^ | 3.07 (0.63)^d^ | 2.79 (0.72)^a^ | 3.20 (0.81)^b^ | *F* (5, 29,823.82) = 78.01*** | | .048 |

*Note. M* = mean; *SD* = standard deviation. Based on the Bonferroni correction, *p* < .003 indicates significant difference between groups. Means significantly differ from others if they have different superscripts.

****p* < .001.

**References**

Arbuckle, J. L. (2016). *SPSS AMOS24 user’s guide*. SPSS Inc.

Byrne, B. M. (2012). *Structural equation modeling with Mplus: Basic concepts, applications, and programming*. Routledge.

Byrne, B. M., Shavelson, R. J., & Muthén, B. (1989). Testing for the equivalence of factor covariance and mean structures: The issue of partial measurement invariance. *Psychological Bulletin*, *105*(3), 456–466. https://doi.org/10.1037/0033-2909.105.3.456

Chen, F. F. (2007). Sensitivity of goodness of ﬁt indexes to lack of measurement invariance. *Structural Equation Modelling*, *14*(3) 464–504. https://doi.org/10.1080/10705510701301834

Enders, C. K. (2010). *Applied missing data analysis*. Guilford Press.

Hill, K. G., White, H. R., Chung, I.-J., Hawkins, J. D., & Catalano, R. F. (2000). Early adult outcomes of adolescent binge drinking: Person- and variable-centered analyses of binge drinking trajectories. *Alcoholism: Clinical and Experimental Research*, *24*(6), 892–901. http://dx.doi.org/10.1111/j.1530-0277.2000.tb02071.x

Lo, Y., Mendell, N. R., & Rubin, D. B. (2001). Testing the number of components in a normal mixture. *Biometrika*, *88*(3), 767–778. https://doi.org/10.1093/biomet/88.3.767

Meeus, W., Van de Schoot, R., Keijsers, L., & Branje, S. (2012). Identity statuses as developmental trajectories: A five-wave longitudinal study in early-to middle and middle-to late-adolescents. *Journal of Youth and Adolescence*, *41*(8) 1008–1021. http://dx.doi.org/10.1007/s10964-011-9730-y

Muthén, L. K., & Muthén, B. O. (1998–2022). *Mplus User’s Guide* (8th ed.). Muthén & Muthén.

Reinecke, J. (2006). Longitudinal analysis of adolescent’s deviant and delinquent behavior. *Methodology*, *2*(3), 100–112. http://dx.doi.org/10.1027/1614-2241.2.3.100

Satorra, A., & Bentler, P. M. (2001). A scaled difference chi-square test statistic for moment structure analysis. *Psychometrika*, *66*(4), 507–514. https://doi.org/10.1007/BF02296192

Schwarz, G. (1978). Estimating the dimension of a model. *The Annals of Statistics*, *6*(2), 461–464. http://dx.doi.org/ 10.1214/aos/1176344136
